# Climate and atmospheric deposition effects on forest water-use efficiency and nitrogen availability across Britain

**DOI:** 10.1038/s41598-020-67562-w

**Published:** 2020-07-24

**Authors:** Rossella Guerrieri, Elena Vanguelova, Rona Pitman, Sue Benham, Michael Perks, James I. L. Morison, Maurizio Mencuccini

**Affiliations:** 1 Centre for Ecological Research and Forestry Applications, CREAF, c/o Universidad Autonoma de Barcelona, Edificio C, 08290 Cerdanyola, Barcelona Spain; 2 Forest Research, Alice Holt Lodge, Farnham, Surrey, GU10 4LH UK; 3 Forest Research, Northern Research Station, Roslin, EH25 9SY Midlothian, Scotland UK; 4 ICREA, Barcelona, Spain; 5 Present Address: Department of Agricultural and Food Sciences, University of Bologna, 40127 Bologna, Italy

**Keywords:** Carbon cycle, Atmospheric chemistry, Ecophysiology, Stable isotope analysis, Environmental monitoring

## Abstract

Rising atmospheric CO_2_ (c_a_) has been shown to increase forest carbon uptake. Yet, whether the c_a_-fertilization effect on forests is modulated by changes in sulphur (S_dep_) and nitrogen (N_dep_) deposition and how N_dep_ affects ecosystem N availability remains unclear. We explored spatial and temporal (over 30-years) changes in tree-ring δ^13^C-derived intrinsic water-use efficiency (iWUE), δ^18^O and δ^15^N for four species in twelve forests across climate and atmospheric deposition gradients in Britain. The increase in iWUE was not uniform across sites and species-specific underlying physiological mechanisms reflected the interactions between climate and atmospheric drivers (oak and Scots pine), but also an age effect (Sitka spruce). Most species showed no significant trends for tree-ring δ^15^N, suggesting no changes in N availability. Increase in iWUE was mostly associated with increase in temperature and decrease in moisture conditions across the South–North gradient and over 30-years. However, when excluding Sitka spruce (to account for age or stand development effects), variations in iWUE were significantly associated with changes in c_a_ and S_dep_. Our data suggest that overall climate had the prevailing effect on changes in iWUE across the investigated sites. Whereas, detection of N_dep_, S_dep_ and c_a_ signals was partially confounded by structural changes during stand development.

## Introduction

The ability of forests to mitigate climate change depends on how well they cope and adapt to the rapid forecasted changes in atmospheric conditions, including pollutant emissions from anthropogenic activities , namely CO_2_, sulphur (S) and reactive nitrogen (N). While sulphur emissions have been successfully regulated in Europe and North America^1^, N compounds, particularly ammonia and nitrous oxide, continue at relatively high levels compared to the pre-industrial period^2–4^. These changes in N and S atmospheric concentrations have occurred together with increasing atmospheric CO_2_ concentration (c_a_), which recently exceeded 410 ppm^5^, with a relative change of 46% compared to pre-industrial levels. Atmospheric N and S compounds are deposited from the atmosphere onto terrestrial ecosystems and together with the increasing c_a_ can have an effect on some of the processes underpinning forest carbon, water and nutrient cycling.

Increases in c_a_ affect leaf gas exchange by increasing photosynthesis (*A*) and reducing stomatal conductance (*g*_*s*_)^6^, thus raising intrinsic water use efficiency of trees (iWUE = *A*/*g*_*s*_). Higher iWUE has been commonly reported for conifer compared to broadleaf species^7^. Moreover, the increase in iWUE derived from carbon isotope composition (δ^13^C) in tree rings^8^ has generally been associated with an active response of trees to increasing c_a_, whereby intercellular CO_2_ (c_i_) increased in a proportional way to c_a_, resulting in a constant c_i_/c_a_ ratio, due to proportional regulation of *A* and *g*_*s*_^9^. However, changes in N (N_dep_) and S (S_dep_) deposition can also influence carbon–water relations. High levels of S_dep_ in the 1980s have been hypothesised to have led to stomata closure^10^, thus current S_dep_ reductions might promote an increase in *g*_*s*_^11^, counteracting the CO_2_-induced water-saving effect of stomatal closure, particularly under non-limiting moisture conditions. Conversely, there is considerable evidence that increased N_dep_ has a fertilization effect on *A*, thereby contributing to enhancing a c_a_ or climate driven increase in tree iWUE^12,13^ and forest C-sinks^14,15^ in N-limited forests.

Atmospheric N represents an additional input of N for trees, particularly for N-limited forest ecosystems in the Northern hemisphere. An increase in N_dep_, however, has been associated with an acceleration of N cycling, with increase in N losses from ecosystems (through nitrate leaching and denitrification), when N saturation is reached^16^. Stable nitrogen isotope composition in plant materials (δ^15^N) has been used as a proxy of changes in ecosystem N availability^17^. An increase in tree δ^15^N has been observed in studies along N_dep_ gradients^18,19^ and soil N manipulation experiments^20,21^. By contrast, a decrease in tree δ^15^N is expected in the case of a reduction of soil N availability (e.g., under a reduction of N_dep_^22^ or in case of tree canopy retention of N_dep_^23^), or when N supply is insufficient to meet the demand caused by CO_2_ fertilization effects on *A*^24^.

Combining multiple isotopes in tree rings across climate and N_dep_ gradients gives a powerful tool to advance our understanding of spatial and temporal patterns of iWUE and its main drivers. In particular, stable oxygen isotope composition (δ^18^O) provides physiological information in addition to that derived from δ^13^C, as its variation in plant material depends on the δ^18^O of the source water and that of the leaf water, the latter affected by transpiration and *g*_*s*_^25^. Moreover, including δ^15^N allows the detection of changes in ecosystem N availability due to changes in the N input from the atmosphere. Studies assessing the effect of N_dep_ and its interactions with other pollutants and climatic variables by using a multiple isotope approach are predominantly site^26^ or species-specific^22^. Analyses at regional scales, and involving different tree species, are paramount to achieving a better understanding of forest functioning in response to global changes.

**Figure 1:**
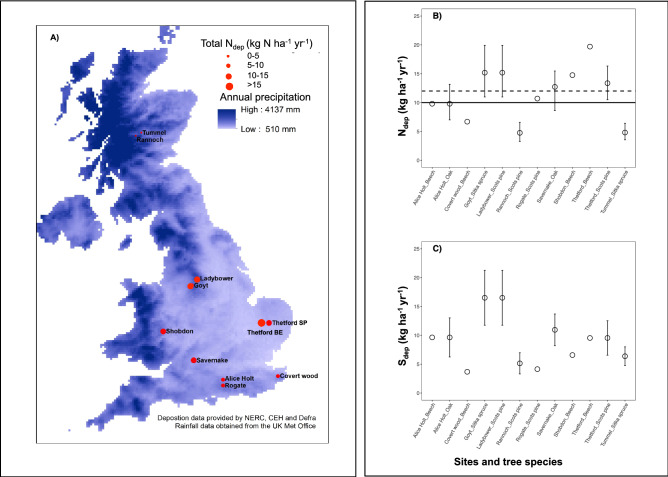
Sites included in the study. Map showing forested sites along the precipitation gradient in Britain (panel **A**) and described in the Table 1. Size of the points reflects the levels of N_dep_, which are reported in the panel **B** for each forest stand and species, together with the S_dep_ (panel **C**). Each point is the average (± standard deviation) of annual values (kg N ha^−1^ year^−1^ and kg S ha^−1^ year^−1^) across 1995–2010 for most of the forest stands, with the exception of Rogate (data from only 2010) and Shobdon and Covert Wood (modelled data, Ref. “Methods”). Note that for Goyt, we considered atmospheric deposition data collected at Ladybower, as the two sites are only 30 km apart. Black solid and dashed lines in the panel B indicate the low and high boundaries for the UK critical load for nitrogen deposition.

We measured δ^13^C_w_, δ^18^O_w_ and δ^15^N_w_ in tree rings from 1980 to 2010 for four of the most common species in Britain, i.e., Scots pine (*Pinus sylvestris* L.), Sitka spruce (*Picea sitchensis* Bong. Carr.), pedunculate oak (*Quercus robur* L.) and European beech (*Fagus sylvatica* L.) in 12 managed forests. Sites were selected along a gradient of climate and N_dep_, while ensuring that within species soil type, forest structure, stand age and management remained similar (Table 1, Fig. 1). Specific goals were to: 1—document the temporal changes and spatial differences in the main climate parameters and N_dep_ and S_dep_ along these South–North gradient in Britain; 2—explore the temporal trends in iWUE, c_i_/c_a_ and oxygen isotope discrimination, Δ^18^O_w_ (i.e., difference between δ^18^O_w_ and precipitation δ^18^O, see “Methods”) at the 12 forests and assess the possible species-specific physiological mechanisms (changes in *A and/or g*_*s*_) underlying variations in iWUE; 3—evaluate whether sites receiving high N_dep_ experience increase in ecosystem N availability and N saturation (using δ^15^N_w_ as a proxy); 4—elucidate the drivers of spatial and temporal changes in the isotope-derived physiological and ecological processes.

**Table 1:** Description of the forest stands included in the study.

Site	Species	Lat	Long	Elevation (m *asl*)	Planting year	BA (m^2^ ha^−1^)	LAI (m^2^ m^−2^)	Soil type	Organic layer C:N	Top soil pH	P_a_ (mm)	T_a_ (°C)
Alice holt	*Fagus sylvatica*	51.1500	− 0.8600	80	1930	34.0	4.17	Eutric Planosol	21	5.1	662	9.7
Thetford	*Fagus sylvatica*	52.4100	0.8700	20	1930	31.0	3.78	Ferralic arenosol	19	3.9	528	9.7
Covert Wood	*Fagus sylvatica*	51.2000	1.1200	20	1950	31.0	5.15	Sceletic Leptosol	20	7.5	527	10.5
Shobdon	*Fagus sylvatica*	52.2400	− 3.0300	200	1952	31.4	5.49	Cambisol	18	4.1	714	9.6
Tummel	*Picea sitchensis*	56.7200	− 4.0500	400	1969	87.9	8.5	Ferric Podzol	37	5.1	796	8.1
Goyt	*Picea sitchensis*	53.2900	− 1.9800	275	1981	59.2	n.a.	Cabric Podzol	22	4.3	697	9.0
Rannoch	*Pinus sylvestris*	56.6500	− 0.4200	470	1965	46.6	6.13	Gleyic Podzol	38	4.2	1161	8.5
Ladybower	*Pinus sylvestris*	53.4100	1.7500	275	1952	49.2	2.93	Cabric Podzol	31	4.1	697	9.0
Rogate	*Pinus sylvestris*	51.0200	− 0.8700	80	1950	37.8 (#)	n.a.	Humic Podzol	25	3.1	662	9.7
Thetford	*Pinus sylvestris*	52.4100	0.8700	20	1967	36.0	4.08	Ferralic Arenosol	25	5.3	528	9.7
Alice Holt	*Quercus robur*	51.1500	− 0.8600	80	1935	24.2	6.1	Eutric Vertisol	19	5.5	662	9.7
Savernake	*Quercus robur*	51.5900	− 1.9200	107	1950	23.1	n.a.	Eutric Vertisol	38	5.2	796	8.1

Main site parameters for the investigated forest stands are given, including Latitude (Lat) and Longitude (Long), stand (planting year, basal area-BA, Leaf Area Index-LAI), soil-related (soil type, C: N ratio and pH) and climate-related (mean of annual precipitation, P_a_, temperature, T_a_ calculated over the investigated years, i.e., 1980–2010) parameters. Basal area was measured in 2010 with the exclusion of Rogate (#), which was measured in 2018. Soil type was classified according to the WRB, 2015^65^. The same climate information was used for Rogate and Alice Holt, as the two sites are very close (within 19 km). This was also the case for Goyt and Ladybower, which are 30 km apart. Note that sites were grouped by tree species to better identify the pairing of sites according to similar age and soil type, but the contrasting levels of N_dep_ (low vs. high nitrogen deposition) are shown in Fig. 1 and Supplementary Table S1. *na* data not available.

## Results

### Spatio-temporal changes in climate and atmospheric deposition across Britain

Across the investigated sites and during the studied years (1980–2010), mean temperature during the growing season significantly increased by approx. 0.02–0.06 °C year^−1^. Whereas, vapour pressure deficit (VPD) did not show a significant trend, except for an increase at the South-eastern most site of Covert Wood (Table 2). The standardised precipitation-evapotranspiration index (SPEI) relative to August, with three months time-scale (SPEI8_3) increased at seven of the sites, but not at Alice Holt, Rogate, Covert Wood, Goyt and Ladybower (Table 2).

**Table 2:** Temporal changes in the main environmental parameters at the investigated sites.

Site	Forest stand	Parameter									
Name	Species	NH_4_ –N	NO_3_-N	Total N_dep_	Total S_dep_	SPEI	T_grs_	T_maxgrs_	P_grs_	VPD_grs_	T_a_
		kg (N or S) ha^−1^ year^−2^				year^−1^	°C year^−1^		cm year^−1^	kPa year^−1^	°C year^−1^
Covert Wood	Beech	n.a	n.a	n.a	n.a	0.03 (0.03)	0.06 (0.01)***	0.08 (0.03)*	0.08 (0.29)	0.008 (0.003)*	0.05 (0.02)*
Alice Holt	Beech	− 0.11 (0.07)	− 0.05 (0.02)	− 0.16 (0.07)*	− 0.28 (0.12)*	0.02 (0.02)	0.02 (0.009)*	0.006 (0.016)	0.008 (0.251)	0.007 (0.003)	0.02 (0.009)*
Alice Holt	Oak										
Rogate	Scots pine										
Savernake	Oak	− 0.4 (0.12)*	− 0.09 (0.04)*	− 0.49 (0.14)**	− 0.2 (0.2)	0.06 (0.02)*	0.03 (0.016)	0.04 (0.03)	0.29 (0.44)	− 0.002 (0.006)	0.03 (0.01)
Thetford	Beech	− 0.29 (0.10)*	− 0.08 (0.03)*	− 0.37 (0.12)**	− 0.36 (0.08)***	0.05 (0.02)*	0.02 (0.008)*	0.03 (0.01)*	0.64 (0.25)*	0.0008	0.02 (0.009)
Thetford	Scots pine										
Shobdon	Beech	n.a	n.a	n.a	n.a	0.06 (0.02)*	0.03 (0.013)*	0.03 (0.02)	0.28 (0.44)	0.005 (0.003)	0.03 (0.01)
Goyt	Sitka spruce	− 0.21 (0.10)	− 0.11 (0.03)**	− 0.31 (0.25)	− 0.52 (0.19)*	0.05 (0.03)	0.03 (0.14)*	0.03 (0.02)	0.68 (0.48)	0.006 (0.004)	− 0.07 (0.01)***
Ladybower	Scots pine										
Tummel	Sitka spruce	− 0.15 (0.07)	− 0.06 (0.05)	− 0.21 (0.10)	− 0.09 (0.11)	0.08 (0.03)*	0.02 (0.01)*	0.03 (0.02)	0.49 (0.43)	− 0.0009 (0.003)	0.02 (0.01)
Rannoch	Scots pine	− 0.15 (0.06)*	− 0.09 (0.03)*	− 0.24 (0.08)*	− 0.3 (0.07)***	0.08 (0.03)*	0.03 (0.010)*	0.02 (0.02)	0.52 (0.53)	0.004 (0.003)	0.02 (0.01)

Values of slope and standard error (in brackets) from linear regression analyses against year of wet NH_4_-N and NO_3_-N, total wet N (NH_4_-N + NO_3_-N) and S deposition, (SO_4_-S), growing season (grs) mean temperature and mean of maximum temperature (T_grs_ and T_maxgrs_), vapour pressure deficit (VPD_grs_) and precipitation (P_grs_), and mean annual temperature (T_a_) for the 12 forest stands across Britain. The same deposition and climate information were used for Rogate and Alice Holt, as the two sites are very close (within 19 km). This was also the case for Goyt and Ladybower, which are 30 km apart. Slopes significantly different from zero were indicated by stars, according to the *p*-values: *p ≤ 0.05; **p ≤ 0.01; ***p ≤ 0.001. *na* long-term data not available.

Principal components analysis (PCA) using across sites long-term averages of environmental variables (PCA_spatial, hereinafter referred to as PCA_s) and within sites annual values (PCA_annual, hereinafter referred to as PCA_a) were conducted to reduce redundancy in the predictors along the climatic gradient. In the case of PCA_s, more than 90% of the variance was explained with two principal components (PC_s). PC_s1 was a general description of site climatic conditions, with highly significant effects from all variables (temperature, VPD and precipitation for the growing season and for the entire year). Strongest (negative) correlations with PC_s1 were with mean and maximum growing season temperature, suggesting that this axis primarily represented a South–North gradient. The PC_s2 correlated most strongly with VPD differences among sites, likely representing the East–West gradient. For the PCA_a, four PCs (PC_a) were required to explain 78% of the total variance. PC_a1 correlated significantly with temporal variability in VPD (negative) and precipitation (positive), PC_a2 with temporal variability in temperature (negative correlations), PC_a3 with growing season (negative) and annual (positive) SPEI indices and VPD (negative), while PC_a4 represented primarily temporal variability in precipitation and VPD (Fig. S1).

Over the 30 year period of the study, the mean annual c_a_ increased by 51 ppm (from 339 to 390 ppm, slope = 1.7 ± 3.9 ppm year^−1^)^27^. Wet N_dep_ and wet S_dep_ decreased between 1995 and 2010 at all sites but Savernake and Tummel for S_dep_, and Goyt/Ladybower and Tummel for N_dep_ (Table 2). Despite the general decreasing trends in wet S_dep_ and N_dep_, half of the investigated sites are above the UK’s critical load for N to woodlands of 10–12 kg N ha^−1^ year^−1^;^28^ (Fig. 1B).

### Species-specific changes in iWUE

Relative changes in iWUE (i.e., value at 2010 minus value at 1980 divided by value at 1980) increased for Scots pine, oak and beech by 17.8 (± 5.6) %, 22.5 (± 11.3) % and 15.3 (± 7.0) %, respectively at all the sites. For Sitka spruce we found instead that iWUE decreased by 22.5 (± 9.9) %, with especially strong reductions in the youngest stand at Goyt that was planted in 1981, when tree-ring δ^13^C time series began (Table S2).

**Figure 2:**
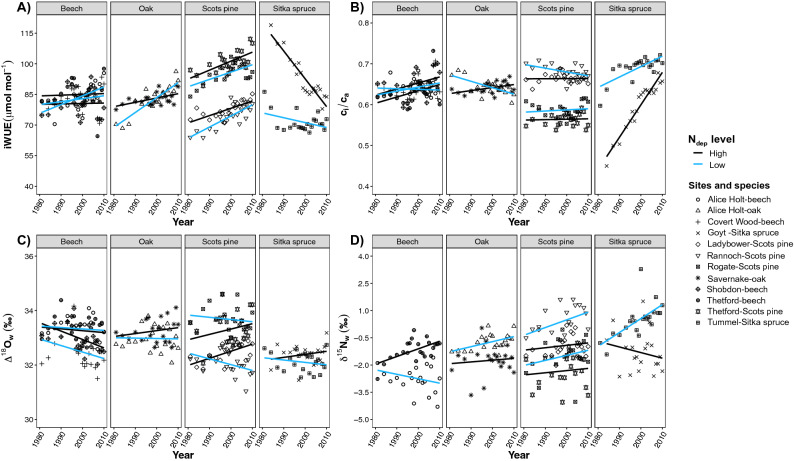
Trends over time in the main physiological and ecological parameters as obtained from the measured tree-ring stable isotopes at the 12 forest stands across Britain. Changes in iWUE (Panel **A**), c_i_/c_a_ ratio (Panel **B**), Δ^18^O_w_ (difference between δ^18^O_w_ and δ^18^O of precipitation, panel **C**) and δ^15^N_w_ (Panel **D**) for four species over the period 1980 to 2010. Each point represents the parameters derived from—or the actual—isotope ratios measured on wood materials pooled from 10 trees for a given year; i.e., δ^13^C_w_—derived iWUE and c_i_/c_a_, δ^18^O_w_—derived Δ^18^O_w_ and measured δ^15^N_w_. Variability among the 10 trees per species and site was assessed for the year 2007 and data are reported in the Table S5. Regression lines are given for each site grouped by Ndep levels (i.e., low Ndep, light blue; high Ndep, black).

Trends in iWUE were not consistent across the investigated species. We found that iWUE increased for Scots pine and oak at all the sites (4 and 2 sites, respectively) over the recent 30 years (Fig. 2A, Table S2). Three of the four beech sites (Covert Wood, Shobdon and Thetford) showed no significant changes in iWUE, while a significant increasing trend in iWUE was observed for beech trees at Alice Holt. In contrast to the other species, the two Sitka spruce stands at Goyt and Tummel showed a reduction in iWUE, particularly for the youngest stand at Goyt (Fig. 2, Table S2).

We tested whether the observed trends could be partially explained by changes in stand parameters i.e., diameter at breast height (DBH), mean height, basal area (BA) for six stands for which data was available (no data were available for the youngest stand at Goyt). Overall we did not find significant relationships between iWUE and stand-related parameters, with the exception of the Scots pine at Ladybower, where iWUE was positively correlated to changes in BA (Supplementary text S1).

### Species-specific trends in c_i_/c_a_ ratio and Δ^18^O_w_

Similarly to what has been reported already for iWUE, changes in the c_i_/c_a_ ratio were not consistent across the species and sites. For both beech and Scots pine at three sites, the parameter did not significantly change over the 30 years (Fig. 2B). The c_i_/c_a_ ratio increased for the Sitka spruce at Goyt (slope = 0.008, *p* < 0.001) and Tummel (slope = 0.003, *p* < 0.001), the oak at Savernake (slope = 0.0007, *p* < 0.05), while it decreased for the oak at Alice Holt (slope = − 0.002, *p* < 0.01) and the Scots pine at Rannoch (slope = − 0.0009, *p* < 0.05).

Trends in Δ^18^O_w_ were not significant, except for the decrease for beech in Shobdon (slope = − 0.03 ± 0.013‰ year^−1^, *p* < 0.05) and for Scots pine at Rannoch (slope = − 0.021 ± 0.009‰ year^−1^, *p* < 0.05) and the increase for Scots pine at Ladybower (slope = 0.026 ± 0.007‰ year^−1^, *p* < 0.01) (Fig. 2C). Changes in Δ^18^O_w_ were not correlated with stand-related parameters, except for Rannoch Scots pine, where we observed a significant negative relationship between Δ^18^O_w_ and mean tree height and BA (Supplementary test S1).

### Spatial and temporal changes in δ^15^N_w_ at different levels of N_dep_

Differences in tree ring δ^15^N_w_ values between sites with N_dep_ below (low N_dep_) and above (high N_dep_) the critical loads (Fig. 1B) did not show a distinctive pattern. In the case of beech, the δ^15^N_w_ values were more positive (on average 1.57‰) at the high N_dep_ than at the low N_dep_ sites (Supplementary Table S3). However, we found the opposite for the oak, Scots pine and Sitka spruce, with trees at higher N_dep_ showing more negative δ^15^N_w_ values (Supplementary Table S3).

No significant trends in δ^15^N_w_ were observed (Fig. 2D), except for oak stands at Alice Holt (slope = 0.03‰ year^−1^, *p* < 0.05), beech stands at Thetford (slope = 0.04‰ year^−1^, *p* < 0.01) and Sitka spruce stand at Tummel (slope = 0.08‰ year^−1^, *p* < 0.01). A significant positive relationship between δ^15^N_w_ and wood %N was observed for 5 sites (Fig. S2), including two beech, one oak and two Scots pine stands, but no relationship was observed in the case of the two Sitka spruce stands.

### Contribution of climate and anthropogenic factors on physiological and ecological processes

**Figure 3:**
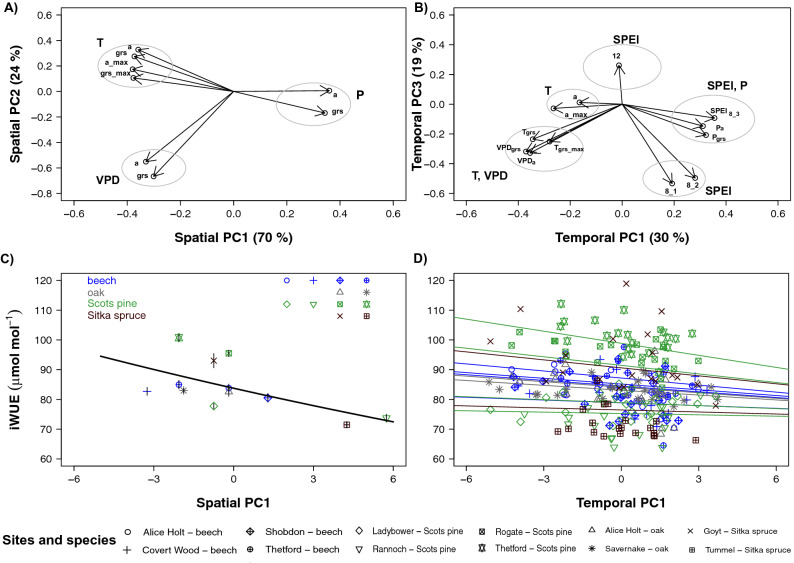
Spatial and temporal changes in iWUE *vs.* climate. Relationship between iWUE (mean through 1980–2010 for each tree species) and PC_s1 (panels **A**–**C**) and between year-by-year iWUE *vs.* PCA_a1 (panels **B**–**D**) across the investigated forest stands as obtained from the linear mixed effect model analyses. For the sake of clarity, we included the results of PCA analyses (panels **A** and **B**), to identify the climate factors that mostly affect the changes in iWUE as shown in the panels **C** and **D**. Slope and standard error values are reported in the Table 4. We used different symbols and colours for regression lines for each species, but slope presented in Table 4 refer to all observations together. The four investigated species were indicated with different colours (see legend in the panel **C** for details), while each site was indicated with a different symbol (see main legend for details). In panels A and B, ‘a’ and ‘grs’ indicate mean annual and growing season T, P and VPD, while 'maxgrs' refers to mean of maximum T. As for the SPEI, 8 and 12 indicate values for the month of August with a time-scale of one (8_1), two (8_2) and three (8_3) months and for the month of December.

One of our goals was to elucidate the relative contribution of climate and atmospheric drivers on changes in physiological and ecological parameters obtained from tree-ring stable isotopes. Temperature increase and precipitation decrease (the dominant parameters in PC_s1) enhanced iWUE along the N-S gradient (Fig. 3A–C, Table 3). Whereas temporal increase in iWUE was mostly associated with changes in parameters related to moisture supply and demand conditions (VPD, SPEI and Precipitation, i.e., the dominant variables in PC_a1 and PC_a3) (Fig. 3B–D, Table 3). However, the high correlations among individual variables in many axes both in the PCA_s and the PCA_a precluded the possibility of drawing definitive conclusions about individual climatic drivers. Nonetheless, neither the increase in c_a_ nor the changes in N_dep_ and S_dep_ were significant predictors in the model (Table 3). The inclusion of stand-related parameters (age and soil type) did not improve the statistical model fit (Supplementary text S2).

**Table 3:** Relationship between physiological and ecological parameters and environmental factors.

Fixed effects	Estimate ± SE	Auto-correlation	Random effect		${\text{R}}_{\text{m}}^{2}$	${\text{R}}_{\text{c}}^{2}$
		Structure	Standard deviation	Residual variance		
**(i) All sites—including c**_**a,**_ **sNdep and sSdep (n = 251)**						
Intercept	80.32 ± 2.30***	0.76	3.82	7.15	0.38	0.52
Conifer versus deciduous	8.26 ± 3.40*					
PCA_s1	− 2.65 ± 0.67*					
PCA_a1	− 0.65 ± 0.12***					
PCA_a3	− 0.40 ± 0.19*					
**(ii) Model (i) without Sitka spruce (n = 210)**						
Intercept	80.75 ± 2.42***	0.50	5.21	4.93	0.41	0.72
Conifer versus deciduous	7.73 ± 3.85 ¥					
PCA_s1	− 2.19 ± 0.79*					
PCA_a1	− 0.64 ± 0.14***					
c_a_	0.20 ± 0.04***					
**(iii) All parameters (n = 140)**						
Intercept	80.94 ± 2.33***	0.54	3.62	4.78	0.66	0.78
Conifer versus deciduous	9.81 ± 3.20*					
PCA_s1	− 4.88 ± 1.12**					
PCA_a1	− 0.67 ± 0.17***					
PCA_a2	− 0.11 ± 0.22 n.s.					
PCA_a3	− 0.46 ± 0.25 ¥					
sNdep	− 0.59 ± 0.65 n.s.					
sSdep	− 1.15 ± 0.45*					
c_a_	0.04 ± 0.09 n.s.					
aSdep	− 0.20 ± 0.19 n.s.					
aNdep	0.11 ± 0.18 n.s.					
**((iv) Model (iii) without the Sitka spruce stands (n = 115)**						
Intercept	81.94 ± 1.86***	0.47	2.85	4.40	0.71	0.80
Conifer versus deciduous	8.51 ± 2.66*					
PCA_s1	− 3.67 ± 0.63**					
PCA_a1	− 0.66 ± 0.17***					
PCA_a3	− 0.52 ± 0.25*					
sS_dep_	− 0.47 ± 0.18*					
aS_dep_	− 1.77 ± 0.40*					

Statistics of the linear mixed effects models for the regression of iWUE as a function of the grouping into conifers and deciduous species (plant functional type, PFT), site (PCA_s) and time (PCA_a) climate variables from PCA analysis, changes in atmospheric CO_2_ (c_a_), spatial (sSdep, sNdep) and annual (aSdep, aNdep) changes in sulphur and nitrogen deposition. Output is given separately for the different model tested: (i) all 12 sites including climate, c_a_ and only sSdep and sNdep; (ii) model (i) excluding the Sitka spruce stands; (iii) all parameters (including aSdep and aNdep, which are available only at 10 sites) and (iv) as model in (iii) but excluding Sitka spruce stands. Estimate of slope and intercept and standard error (SE) values are provided for each of the fixed factors. We considered random intercept for the site × species combination. Marginal (only fixed factors) and conditional (fixed + random factors) proportions of the explained variance are indicated as R^2^_m_ and R^2^_c_, respectively. For each model, the number of observations is indicated with ‘n’. Note that we report results from the final model (Ref. “Methods”), but all the different models tested are listed in the Table S7 and results reported in the supplementary text S4. All independent variables were centered prior to analysis. Significance levels for fixed factors referring to simultaneous tests for general linear hypothesis testing are indicated as follows: ¥, *p* ≤ 0.10; *, *p* ≤ 0.05; **, *p* ≤ 0.01; ***, *p* ≤ 0.001.

Given that the iWUE trend observed for the Sitka spruce stands could be partially related to the age effect, with particular reference to the youngest one at Goyt, we repeated the analyses excluding Sitka spruce. Results confirmed no relationship between N_dep_ and iWUE (Table 3). We found, however, a negative relationship between both sS_dep_ and aS_dep_ and iWUE (Table 3) and the expected significant relationship between c_a_ and iWUE (Table 3). Finally, when performing the analysis by level of N_dep_ (i.e., high and low N_dep_ for sites above and below the critical loads, respectively) and by including or not the Sitka spruce stands, the positive relationship between iWUE and c_a_ turned out to be significant, with the exception of the analysis for the high N_dep_ sites and when the Sitka spruce stand at Goyt was included (Supplementary Table S4).

Differences in both spatial and temporal changes in climatic conditions significantly affected Δ^18^O_w_. Three axes from the PCA_a analyses (PC_a1, PC_a2 and PC_a3) were significant, suggesting a combined effect of temperature and moisture variability on changes in Δ^18^O_w_ (Table 4). When considering the subset of sites where annual atmospheric deposition data were available, we did find a significant and negative relationship between Δ^18^O_w_ and sS_dep_ and aS_dep_. This, however, was not the case when Sitka spruce stands were removed from the analyses, to account for possible age effects on changes in Δ^18^O_w_ (Table 4, Supplementary text S5).

**Table 4:** Relationship between physiological and ecological parameters and environmental factors.

Fixed effects	Estimate ± SE	Auto-correlation	Random effect		${\text{R}}_{\text{m}}^{2}$	${\text{R}}_{\text{c}}^{2}$
		Structure	Standard deviation	Residual variance		
**Δ**^**18**^**O**_**w**_**—(i) All sites including c**_**a**_**, sSdep, sNdep (n = 251)**						
Intercept	32.88 ± 0.18***	0.25	0.38	0.41	0.31	0.63
Conifer versus deciduous	− 0.06 ± 0.27 n.s.					
PCA_s1	− 0.12 ± 0.05*					
PCA_a1	− 0.05 ± 0.013***					
PCA_a2	0.11 ± 0.016***					
PCA_a3	0.05 ± 0.02**					
sSdep	− 0.03 ± 0.03 n.s.					
**Δ**^**18**^**O**_**w**_**—(ii) All parameters, 10 sites (n = 140)**						
Intercept	33.07 ± 0.08***	0.12	0.11	0.35	0.72	0.75
Conifer versus deciduous	− 0.05 ± 0.01 n.s.					
PCA_s1	− 0.24 ± 0.02***					
PCA_a1	− 0.06 ± 0.01***					
PCA_a2	0.12 ± 0.02***					
PCA_a3	0.05 ± 0.11*					
sSdep	− 0.10 ± 0.01***					
aSdep	− 0.03 ± 0.01*					
**Δ**^**18**^**O**_**w**_**—(iii) All parameters and sites without Sitka spruce (n = 210)**						
Intercept	33.03 ± 0.19***	0.27	0.45	0.44	0.10	0.56
Conifer versus deciduous	− 0.16 ± 0.30 n.s.					
PCA_a1	− 0.05 ± 0.01***					
PCA_a2	0.10 ± 0.02***					
**Δ**^**18**^**O**_**w**_**—Climate and N**_**dep**_ **10 sites (n = 204)**						
Intercept	− 1.25 ± 0.27***	0.14	0.46	0.82	0.43	0.57
Conifer versus deciduous	− 0.04 ± 0.37 n.s.					
sN_dep_	0.14 ± 0.07 ¥					
PCA_s1	0.51 ± 0.13**					

Statistics of the linear mixed effects models for the regression of for Δ^18^O_w_ and δ^15^N_w_ as a function of the grouping into conifers and deciduous species (plant functional type, PFT), site (PCA_s) and time (PCA_a) climate variables from PCA analysis, spatial (sSdep, sNdep) and annual (aSdep, aNdep) changes in sulphur and nitrogen deposition. For Δ^18^O_w_ output is given separately for the different model tested (Ref. “Methods”): (i) all 12 sites including climate, c_a_ and only sSdep and sNdep; (ii) all parameters (including aSdep and aNdep, which are available only at 10 sites) and (iii) models (i–ii) without Sitka spruce stands (both leading to the iii) as final model (Ref. Table S7 and Supplementary text S5). Estimate of slope and intercept and standard error (SE) values are provided for each of the fixed factors. We considered random intercept for the site × species combination. Marginal (only fixed factors) and conditional (fixed + random factors) proportions of the explained variance are indicated as R^2^_m_ and R^2^_c_, respectively. For each model, the number of observations is indicated with ‘n’. Note that we report results from the final model, but all the different models tested are listed in the Table S7 and results reported in the supplementary text S5 and S6. All independent variables were centered prior to analysis. Significance levels for fixed factors referring to significance in simultaneous tests for general linear hypothesis testing are indicated as follows: ¥, *p* ≤ 0.10; *, *p* ≤ 0.05; **, *p* ≤ 0.01; ***, *p* ≤ 0.001.

When considering all sites and species together, at high and low N_dep_ sites, a clear pattern for δ^15^N_w_ in tree rings was not seen and no differences were observed when grouping species by plant functional type (Table 4). Changes in δ^15^N_w_ were explained primarily by climate factors, particularly temperature and precipitation, with a marginal (*p* = 0.1) effect of differences across sites in N_dep_ (Table 4). Soil type and stand age did not appear significant predictors for δ^15^N_w_ in the statistical model (Supplementary text S3).

**Figure 4:**
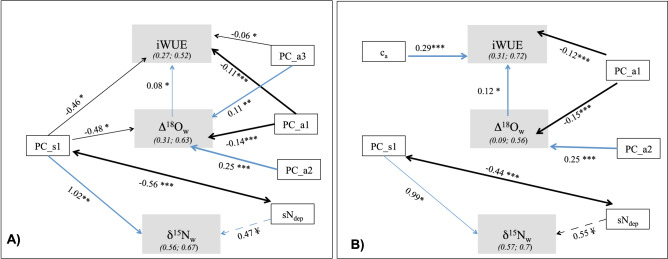
Direct and indirect effects on tree-ring stable isotopes and derived physiological parameters. Result from the structural equation modelling analysis, which included all sites (panel **A**) and all sites excluding the two Sitka spruce stands (panel **B**). Details of equations are reported in the Table S7. Continuous arrows indicate relationships significant at the *p* ≤ 0.05 or greater (depending on number of stars, i.e., *p ≤ 0.05; **p ≤ 0.01; ***p ≤ 0.001). Dashed arrows (and the ‘¥’ symbol) indicate relationships significant at *p* ≤ 0.10. Thickness of lines reflects level of significance. Notice that degrees of freedom vary between site-based and time-based analyses. Black and blue arrows indicate negative and positive relationships, respectively. Double-headed arrow indicates correlated errors between variables. PC_s1 indicates the first component from the PCA_s (using long-term average climate variables across sites) and PC_a1, PC_2 and PC_a3 the first three principal components from the PCA_a (using annual climate parameters). Number next to the paths indicate standardized path coefficients. Numbers below each isotope-related parameters indicate marginal and conditional R^2^, respectively. They are not always the same as those reported in the Tables 3 and 4 due to slightly different equations considered in the SEM analyses. In particular, PFT was not included as fixed factor in any model, while Δ^18^O_w_ was included as fixed factor in the model for iWUE (Ref. Table S7 for all details).

The combined influences of temporal changes and spatial differences in climatic and atmospheric deposition variables across all three isotopes were examined with the help of a mixed-effect path model (Fig. 4A). Consistent with the earlier analysis using linear mixed models, the first axis of PC_s significantly affected all three isotopes, negatively for iWUE and Δ^18^O_w_ and positively for δ^15^N_w_. This is consistent with the first axis of PC_s being related to a South–North gradient (i.e., positively with precipitation and negatively with temperature and VPD). Conversely, the first and third axis of PC_a only affected iWUE (both effects negative) and Δ^18^O_w_ (negative effect for PC_a1 and positive effect for PC_a3). This is also consistent with PC_a1 being positively related to precipitation and SPEI and negatively related to VPD and temperature. N_dep_ was only marginally (*p* < 0.1) important for δ^15^N_w_. A significant positive effect of Δ^18^O_w_ on iWUE was also found, while no relationship was observed between iWUE and δ^15^N_w_ (Fig. 4A). Similar results were found when excluding Sitka spruce stands from the analyses for both Δ^18^O_w_ and δ^15^N_w_, in addition to the significant positive effect of c_a_ on iWUE and (Fig. 4B). When including in the analyses only sites where annual atmospheric deposition data were available, we also found a negative effect of sS_dep_ and aS_dep_ on iWUE (Fig. S5).

## Discussion

### Climate and atmospheric deposition changes across the investigated sites

The first goal of this study was to document the changes in climate and atmospheric deposition across the investigated sites over the period 1980–2010, so as to better assess their effects on iWUE and other isotope-related parameters. Trees at most of the investigated sites experienced increasingly wetter and warmer growing conditions, particularly in Scotland, corroborating general trends for Britain^29^. PC analyses showed that temperature and precipitation are the main climate factors describing the South–North gradient, while parameters related moisture conditions (SPEI, VPD and precipitation) were more relevant in long-term climate variations.

While c_a_ has continued to increase by 14% from 2010 to 1980, differences were observed in magnitude and directionality of changes in atmospheric deposition. A significant reduction of total wet S_dep_ and N_dep_ (NH_4_-N + NO_3_-N) was also observed at most sites^1,30,31^, with steeper slopes for S_dep_ than N_dep_. Decreasing S_dep_ is consistent with national scale reports, whereby emissions and total S_dep_ have greatly declined over the last few decades^4^. However, a significant decline in total N_dep_ (including oxidised and reduced form) has not yet been reported nationally despite reduction of N emissions of both nitrogen oxides and ammonia^4^. Taken all together, these results suggest that directionality and magnitude of changes in N_dep_ and S_dep_ across the environmental gradient may modulate tree species response to c_a_, as we discuss in the following paragraphs.

### Tree species differed in iWUE trends and underlying mechanisms

Our second goal was to assess trends in iWUE and elucidate potential physiological mechanisms underlying changes in iWUE. Three of the four investigated tree species showed that iWUE increased over 30-years, corroborating global tree-ring δ^13^C based analyses^12,13,32^. Across sites, the relationship between iWUE and Δ^18^O_w_ (Fig. 4) suggests that overall there is a tight coupling between *A* and *g*_*s*_ across the investigated species. However, the within-sites relationships were significant only at 5 of the 12 forest stands (Table S2), suggesting physiological strategies differ amongst—and likely also within—tree species.

Scots pine and oak showed an increase in iWUE, but the underlying mechanisms were likely different. Scots pine at southernmost sites (Rogate and Thetford) showed no changes in transpiration losses (no significant trend for Δ^18^O_w_) and a proportional regulation of *A* and *g*_*s*,_ which contributed to a constant c_i_/c_a_. Scots pine has a very conservative strategy regarding water use, due to its tight stomatal control under moisture limitation^33^. Whereas, Δ^18^O_w_ for Scots pine at the northernmost sites (Ladybower and Rannoch) significantly changed, though in the opposite direction. The increase in Δ^18^O_w_ for Scots pine at Ladybower would indicate a reduction in g_s_, which is supported by the positive relationship between iWUE and Δ^18^O_w_ (Table S2). The site showed the highest mean S_dep_ values among all sites. Even though trend in S_dep_ has significantly decreased at the site, we cannot exclude a possible legacy (negative) effect of deposition on g_s_^23^. In the case of Rannoch, the physiological signal recorded in the reduction in Δ^18^O_w_ could be partially confounded by tree stand development (as a negative relationship was found between Δ^18^O_w_ and tree height and BA) or the significant reduction in S_dep_, which would have a positive effect on g_s_^10^.

In contrast, the increase in iWUE and the significant changes in the c_i_/c_a_ ratio (though in opposite direction) for the two oak stands was mostly related to a more dynamic adjustment of leaf gas exchanges to environmental conditions, with likely reductions in *g*_*s*_ and lower *A*, leading to a lower increase in iWUE in the case of Savernake (where both c_i_/c_a_ and Δ^18^O_w_ increased) compared to Alice Holt (no changes in *g*_*s*_*,* as suggested by no significant trend in Δ^18^O_w_, while c_i_/c_a_ decreased). This result indicated that S_dep_ is still negatively affecting oak trees at Savernake—one of the few sites where S_dep_ has not significantly declined over the last decades. Consistent with our results, a previous study found that exposure to high SO_2_ pollution significantly reduced g_s_ leading to an increase in iWUE for oak trees in southern England^10^.

The reduction in iWUE in the fast-growing^34^ Sitka spruce trees, particularly the youngest stand at Goyt), most likely reflect age or management history effects. Height growth during tree development may affect δ^13^C and hence iWUE estimates via changes in light availability^35^ and hydraulic conductance^36^, but also through increasing LAI as canopy develops and contributes to respired CO_2_^37^. Note, however, that the trend in iWUE at Goyt cannot be explained by increasing hydraulic constraints with tree height^36^, which would be in the opposite direction (i.e., an increase in iWUE in taller trees). It is most likely that the observed trends may reflect common historical upland conifer establishment practices, e.g., fertilisation on tree establishment until approximately 10 years of age and thinning between 15 and 20 years. This may have contributed to reduce tree competitions for light and soil water, thus accelerating growth but reducing iWUE in the early stage of development until canopy closure^38^.

Beech was the least responsive species, as no changes in iWUE were observed in general, even though the four selected sites were along a clear precipitation gradient (Fig. 1). However, the wettest site (Shobdon) showed the lowest % increase in iWUE (only 5% compared to 16–19% changes at the remaining three sites), which could partially be explained by the increase in g_s_ suggested by the reduction in Δ^18^O_w_. Moreover, when comparing even-aged beech and oak stands at the same site (i.e., Alice Holt), with similar soil and climate conditions, we found that despite the two species showing similar iWUE, beech had a higher Δ^18^O_w_ than oak (Fig. S3). This suggests differences between the two species for g_s_, which could partially explain the significantly (*p* < 0.05) higher slope in beech compared to oak for temporal trends in Δ^18^O_w_.

### Changes in tree iWUE and Δ^18^O_w_ across Britain were associated with climate and atmospheric S deposition

Changes (over time) and differences (across sites) in moisture conditions and temperature along the North–South gradient in Britain were overall more important than atmospheric deposition in explaining variations in iWUE. Increase in temperature, both moving North–South (PC_s1) across Britain and over time (PC_a2), significantly increased iWUE. This could be explained by a positive effect of temperature on *A*, but also a reduction in g_s_, likely associated with the increase in VPD, the major component of PC_a3. Indeed, the latter was significantly and positively correlated with Δ^18^O_w_. This finding is in agreement with results for two broadleaf species in the Northeastern US^26^, whereby changes in iWUE were predominantly associated with soil moisture conditions and no effects of either N_dep_ or S_dep_ were observed.

The detection of atmospheric deposition and c_a_ signals in our study, however, was possibly confounded by likely age effects and/or associated tree structural and functional changes (as we discussed above). Interestingly, when Sitka spruce stands were excluded from the analyses, the increase in iWUE was associated with rising c_a_, but also with the reduction in S_dep_, while there was no relationship with N_dep_. This is quite important, as S_dep_ and c_a_ may have a similar effect on g_s_, i.e., an increase in S_dep_ and c_a_ leads to a reduction in g_s_^10^. However, changes in S_dep_ and c_a_ do not always follow the same direction. Indeed we reported a reduction in S_dep_ at most of the sites, under a general increase in c_a_. The negative relationship between Δ^18^O_w_ and S_dep_ suggests that the alleviation of the negative effects from S_dep_ on g_s_^22^ may be stronger than the c_a_ effect leading to stomatal closure (which does not find indication in our data). These results on one hand suggest that variations in S_dep_ outweigh changes in N_dep_ and c_a_. S_dep_ may influence tree water-use directly, by affecting g_s_^22^ or indirectly, by leading to soil acidification, loss of calcium, which play a significant role in controlling g_s_^39^. On the other hand, they indicate that it is essential to account for changes caused by stand development and management history, if the goal is to disentangle climatic and anthropogenic drivers of change in iWUE.

### No changes in ecosystem N availability due to N_dep_

Given that some of the investigated forest stands have reached the critical load in terms of N_dep_ (Ref. Fig. 1B), we expected to find significant changes in ecosystem N due to possible N losses from the forest ecosystems. Yet, spatial and temporal variations in δ^15^N_w_ values did not indicate differences in ecosystem N dynamics between high and low N_dep_ levels. If this was the case, we should have observed an increase in tree-ring δ^15^N_w_ over time and moving from low to high N_dep_ sites, as a consequence of high nitrification and loss (through denitrification and/or leaching) of the ^15^ N-depleted NO_3_, leaving trees with ^15^ N-enriched NO_3_^40^. This was only observed for the beech at Thetford, which is already confirmed as an N saturated site by detailed gradient studies^41^. Repeated soil analysis suggests N accumulation in soil organic layers under the conifer species, including the Tummel Sitka spruce site over the last 15 years, but no signs of N saturation^41^. However, a significant positive relationship has been observed between N_dep_ and NO_3_ leaching across some conifer sites in this study, so further N input to conifer forests could cause significantly higher NO_3_ leaching^41^.

Our tree-ring δ^15^N_w_ data suggest that N availability has not changed for most of the forest stands over the investigated 30-years period, which is in contrast to declining δ^15^N trends reported both in the USA^22,42^ and globally^43^, whereby studies suggest on going oligotrophication. However, we cannot exclude that N recycling processes within trees could also have contributed to the observed lack of a trend in δ^15^N_w_^44^, particularly in the case where no relationship was found between δ^15^N_w_ and wood %N (Fig. S1).

The strong climate signal explaining spatial variations in the tree-ring δ^15^N values is consistent with a global analysis^45^, which identified temperature and precipitation as the main climatic drivers of changes in the foliar δ^15^N_w_ across different forest ecosystems. An indirect effect of wet N_dep_-via climate differences- on tree-rings δ^15^N_w_ cannot be fully excluded. Indeed, the significant negative relationship between atmospheric deposition and PC_s1, partially indicating that wet N_dep_ is strongly related to the amount of precipitation. The predominantly westerly airflow across the UK brings less polluted air from the Atlantic. Orographic cloud formation in the more mountainous regions of the NW however leads to a substantially higher rainfall and consequently higher N_dep_^46^.

Our results also indicate that pinpointing variations in δ^15^N_w_ caused by gradual changes in ambient N_dep_ is more challenging than in N manipulation experiments, where abrupt and high N doses over relatively short periods of time predominate. Moreover, the lack of information regarding both temporal variability in dry N_dep_ and changes in the N isotopic signatures for NH_4_ and NO_3_ might increase the uncertainties in the detection of changes in atmospheric N_dep_ by using δ^15^N in tree rings^47^. Monitoring changes in the isotopic composition of N-specific compounds in rainfall over time can greatly improve our ability to use δ^15^N in tree rings to detect changes in N input from N_dep_.

## Conclusions

Long-term forest monitoring systems, such as the Level II—ICP forests programme, provide unique near-natural systems for assessing the effect of climate change on ecophysiological responses of different tree-species at a regional scale and elucidating interactions among environmental forcing factors and forest ecosystem response. Our results showed that the increase in iWUE was not uniform across sites and that species-specific underlying physiological mechanisms were likely affected by the interactions between climate and atmospheric drivers (for oak and Scots pine), but also an tree structural changes during stand development (for Sitka spruce).

Spatial and temporal changes in temperature and moisture conditions overrode the effect of atmospheric deposition and c_a_ on changes in iWUE for the investigated forests in Britain. This is remarkable since such an increase is widely predicted to occur in response to increasing c_a_. Our results suggest that the effect of increasing c_a_ on temporal changes in iWUE could be over estimated, if concomitant changes in atmospheric deposition or ontogenic effects (such as structural changes during tree development) are not accounted for. The tree-ring δ^15^N_w_ analyses did not provide evidence for changes in N availability caused by changes in N_dep_. In particular, sites receiving high N_dep_ (now considered above the critical loads) did not show evidence of N saturation, with the exception of the beech site at Thetford. Spatial differences in tree ring δ^15^N_w_ were mostly explained by differences across sites in temperature and precipitation, rather than changes in N_dep_.

The multiple-species and regional analysis indicate that climate change may affect the most common native and introduced species in British woodlands. Lower summer rainfall and high temperature and VPD are likely to become more frequent in South-eastern Britain, thus affecting the future site suitability of beech woodland, as the species is more susceptible to drought^48^, while oak and Scots pine could cope better (see e.g., results at Alice Holt). However, even though oak may be physiologically plastic in response to future climate change, widespread oak decline across Britain has been observed related to a number of biotic and abiotic factors, including climate and pollution^49^. Sitka spruce, the major upland planted timber species in the UK, native of coastal Northwestern America, is likely to maintain continued good growth in British northern uplands where water stress is less pronounced. However, the young ages of these stands and intensity and frequency of management interventions make it more difficult to disentangle development from environmental effects on iWUE trends.

## Methods

### Site and sampling

We selected twelve monoculture tree stands of the most common tree species in Britain, Scots pine (*Pinus sylvestris* L.), Sitka spruce (*Picea sitchensis* Bong. Carr.), pedunculate oak (*Quercus robur* L.) and common beech (*Fagus sylvatica* L.). The majority of the stands were experimental sites within the Level II- ICP intensive forest monitoring network (http://icp-forests.net/), with the exception of Covert Wood, Shobdon and Goyt. The Goyt site was added as a high N_dep_ site as a contrast to the low N_dep_ Sitka spruce site in Scotland (Fig. 1, Table 1, Supplementary Table 1). For each species, forests were selected with similar soil type and age, but with contrasting N_dep_, S_dep_ and climate, particularly rainfall and temperature, as described in Fig. 1, Table 1 and Supplementary Table 1. Stand information (mean tree height, mean diameter at the breast height and basal area) as measured for target years and for some of the forest stands are shown in Fig. S4.

At each ICP forest site, a plot of 0.25 ha was established in 1995 to carry out monitoring^30^ and a similar protocol was followed at the Goyt and Shobdon sites. Within each plot, 10 trees were selected for the collection of 3 wood cores per tree by using a 5 mm diameter increment borer, which were placed in paper straws for transport. Sampling was carried out between November 2010 and March 2011. Once in the laboratory, samples were dried at 70 °C for 48 h. Of the three wood cores sampled, one was kept for dendrochronology, with the other two used for stable isotope analyses.

### Climate and atmospheric deposition data

Climate data (Temperature, T, Vapour Pressure Deficit, VPD, Precipitation, P) were obtained from automated weather stations at the sites and/or the nearest local meteorological stations (data were provided by the British Atmospheric Data Centre). Annual mean (T_a_) and mean maximum (T_amax_) values for temperature were calculated from monthly mean and maximum air temperature, T, respectively, and annual precipitation (P_a_) was calculated as the sum of total monthly precipitations. Annual VPD was calculated from averaging monthly values obtained from mean monthly maximum temperature and minimum monthly relative humidity. For all the parameters, mean values were also calculated over the growing season, i.e., from May to September. We also considered the standardised precipitation-evapo-transpiration index, SPEI, relative to August, with 1 (SPEI8_1), 2 (SPEI8_2) and 3 (SPEI8_3) months time-scale and SPEI relative to December, with 1 and 12 months time-scale, the latter providing year-cumulated soil moisture conditions. SPEI values were obtained from the global database with 0.5 degrees spatial resolution available online at: https://sac.csic.es/spei/.

Yearly wet nitrogen (N_dep_) and sulphur deposition (S_dep_) were obtained from measured bulk precipitation and throughfall water volumes at the sites and measured elemental concentrations (NO_3_^−^, NH_4_^+^ and SO^2–^_4_) as previously described^30^. For the spatial analyses, we considered mean of annual deposition (sN_dep_ and sS_dep_), obtained as the sum of total (NH_4_-N + NO_3_-N for N_dep_) wet and dry deposition. The latter were estimated as difference between throughfall and bulk precipitation N fluxes^30^. For Rogate only 1 year (2010) of monitoring was available. For Goyt site, atmospheric deposition data collected at Ladybower were considered, as the two sites are 30 km apart. For two sites (i.e., Shobdon and Covert Wood), which were not part of the regular ICP forest sites, the wet deposition obtained from the UK 5 × 5 km grid N_dep_ and S_dep_ dataset was used^4^. The estimate included wet and dry NH_x_-N (NH_4_, NH_3_), NO_y_-N (NO_2_, NO_3_, HNO_3_) and S (SO_x_ = SO_2_ and SO_4_) deposition, modelled using FRAME with 2005 emissions data^4^. However, only the total wet deposition was included in the analyses here, as we previously reported a good agreement between modelled and measured wet N_dep_^50^.

For the temporal analyses, only wet deposition (as calculated from NO_3_^−^, NH_4_^+^ and SO^2–^_4_ concentrations in bulk precipitation) was considered (indicated as aN_dep_ and aS_dep_), given the uncertainties associated with the quantification of the dry deposition. For instance, when differences between throughfall and bulk precipitation are < 0 it is assumed atmospheric deposition is retained by tree canopies, but this does not necessarily mean that there is no dry deposition. At Rogate only one-year data were available so we considered annual wet deposition data for Alice Holt, which is within 19 km distance. This was also the case for Goyt and Ladybower, which are 30 km apart. Shobdon and Covert Wood were not included in the analyses where annual deposition data were considered (see earlier in the text and Ref. Table S7).

### Stable isotope analyses

Wood cores were subjected to removal of mobile N and extractives with a soxhlet apparatus as described in Guerrieri et al.^23^. After the chemical pre-treatment, wood cores were dated and cross-dated from 2010 back to 1980 and then separated with a scalpel as follow: single annual rings from 2010 to 1995 and then groups of 3 annual rings from 1994 back to 1980. We maintained the annual resolution from 1995 onward because this is the period when the UK-ICP forest network was established and atmospheric deposition was monitored.

To minimise the cost of the stable isotope analyses while including 12 sites and 4 different tree species, the wood materials were pooled from 10 trees (2 wood cores per tree) for each given ring or group of rings. However, for one year (i.e., 2007) and at two sites for each species, carbon and oxygen isotope ratios for each of the 10 trees was measured, so as to assess the variability among trees (Supplementary Table S5). For each core per tree species, each ring was cut with a razor blade under a microscope, milled and homogenized in a centrifugal mill, and then pooled by year. Moreover, for δ^15^N_w_ analyses, we only included the sites where long-term atmospheric deposition data were available (i.e., tree species at Shobdon and Covert Wood were excluded).

An amount of 0.4–0.6 mg of extracted wood sample from each given ring (or group of annual rings for the years 1994 back to 1980) was weighed in tin capsules and combusted in the elemental analyzer (NA2500, Carlo Erba) for the determination of δ^13^C_w_ by VG Prism III Isotope ratio mass spectrometer at the School of Geosciences (University of Edinburgh, UK). For δ^15^N_w_, 25–30 mg of wood sample was weighed in tin capsules and combusted on a PDZ Europa ANCA-GSL elemental analyzer interfaced to a PDZ Europa 20–20 isotope ratio mass spectrometer (Sercon Ltd., Cheshire, UK). For δ^18^O_w_, 0.8–1 mg of each sample was weighed in silver capsules and analyzed on a PyroCube (Elementar Analysensysteme GmbH, Hanau, Germany) interfaced to an Isoprime VisION (Isoprime Ltd., Stockport, UK, a unit of Elementar Analysensysteme GmbH, Hanau, Germany). Analyses were carried out at the Stable isotopes facilities of the School of GeoSciences (University of Edinburgh, UK) for δ^13^C_w_, and at the Stable Isotope facility of the UC Davis, University of California (USA) for δ^18^O_w_ and δ^15^N_w_. Stable isotope abundances are expressed as ratios of ^13^C/^12^C, ^15^ N/^14^ N and ^18^O/^16^O using δ-notation (in per-mil;‰) relative to international standards (VPDB for δ^13^C_w_, atmospheric N_2_ for δ^15^N_w_ and VSMOW for δ^18^O_w_). The standard deviation for internal standards was 0.1‰ for δ^13^C_w_ (PACS-2), 0.2, 0.3 and 0.4‰ for δ^18^O_w_ (IAEA 600, IAEA 601 and IAEA 602, respectively), and between 0.1 and 0.3‰ for δ^15^N_w_ (USGS-41 Glutamic Acid and peach leaves, respectively).

### Calculation of iWUE and Δ^18^O_w_

The iWUE and the c_i_/c_a_ ratio were derived from measured δ^13^C_w_ values, and based on the well-established theory linking leaf c_i_/c_a_ with carbon isotope discrimination, Δ^13^C_w_,^51^ as shown in the equation below:

1 $$ \Delta^{13} C_{w} = a + \left( {b - a} \right) \frac{{c_{i} }}{{c_{a} }} = \frac{{\left( {\delta^{13} C_{a} - \delta^{13} C_{w} } \right)}}{{\left( { 1 + \frac{{\delta^{13} C_{w} }}{1000}} \right)}} $$

where δ^13^C_a_ and δ^13^C_w_ are the carbon isotope compositions of c_a_ and wood, *a* is the isotope fractionation during CO_2_ diffusion through stomata (4.4‰) and *b* is the isotope fractionation during fixation by Rubisco (27‰). Note that Eq. (1) is the “simplified version” of the Farquhar model describing carbon isotope discrimination in plant material, which does not include effects due to mesophyll conductance and photorespiration. We derived c_i_ from the following equation:

2 $$ c_{i} = c_{a} \frac{{(\delta^{13} C_{a} - \delta^{13} C_{w} ) - a}}{b - a} $$

c_a_ values were obtained from Mauna Loa records^27^, and δ^13^C_a_ values were obtained from Mauna Loa records^5^ from 1990 to 2010, while from 1990 back to 1980 we used data published in Ref.^52^. iWUE (μmol CO_2_ mol^−1^ H_2_O) was then calculated using the following equation:

3 $$ iWUE = \frac{A}{{g_{s} }} = \frac{{c_{a} - c_{i} }}{1.6} = \frac{{c_{a} }}{1.6} \left( {\frac{{b - \Delta^{13} C_{w} }}{b - a}} \right) $$

where 1.6 is the molar diffusivity ratio of CO_2_ to H_2_O (i.e., g_CO2_ = g_H2O_/1.6). Note that in the Eqs. (2) and (3) we used average of values measured over growing season months (May–September) for both c_a_ and δ^13^C_a_.

Tree-ring oxygen isotope discrimination, Δ^18^O_w_, was calculated according to Eq. (4)^53^:

4 $$ \Delta^{18 } O_{w} = \frac{{\delta^{18} O_{w} - \delta^{18} O_{s} }}{{1 + \left( {\frac{{\delta^{18} O_{s} }}{1000}} \right)}} $$

where δ^18^O_w_ is the oxygen isotope composition we measured in each ring, while δ^18^O_s_ is the oxygen isotope composition of the source water, i.e. the soil water, which we assumed to reflect the δ^18^O of precipitation (δ^18^O_P_). We estimated annual values of δ^18^O_P_ at each site as described by Barbour et al.^54^, by considering the following equation:

5 $$ \delta^{18} O_{P} = 0.52T_{a } - 0.006T_{a}^{2} + 2.42P_{a} - 1.43P_{a}^{2} - 0.046\sqrt E - 13.0 $$

where T_a_, P_a_ and E are the annual mean air temperature, precipitation (this latter expressed in m) and elevation (m *asl*), respectively. Mean values of estimated δ^18^O_P_ from Eq. (5) were in line with estimates from the Online Isotopes in Precipitation Calculator (https://wateriso.utah.edu/waterisotopes/pages/data_access/oipc.html) and measured δ^18^O_P_ values at Keyworth (Supplementary Table S6). The modelled δ^18^O_P_ did not show a significant trend at most of the sites, with the exception of Goyt/Ladybower (slope = − 0.03 ± 0.007‰ per year, *p* < 0.001) and Covert wood (slope = 0.02 ± 0.009‰ per year, *p* = 0.05).

We assumed Δ^18^O_w_ to reflect the leaf water Δ^18^O, which is affected by transpiration. Notably, less enriched (in ^18^O) water from the soil and more enriched (in ^18^O) water at the leaf evaporative sites continuously mix, as a function of transpiration rates and the pathway of water movement through foliar tissues (i.e., Péclet effect)^55^ so that lower leaf Δ^18^O results from an increase in transpiration and g_s_^56^.

The physiological signal imprinted in the foliage may be dampened in tree rings, due to post-photosynthetic fractionation during translocation of sucrose and synthesis of cellulose in the tree stem^57^. This leads to an offset between foliar and tree-ring δ^18^O and also δ^13^C values. However, accounting for the offset when interpreting tree-ring isotopes is still challenging, as it is not clear whether the offset is species-specific, if it is maintained over the long-term and what are the mechanisms driving it^57,58^.

### Statistical analyses

Linear regression analyses were initially used to explore whether (1) temporal trends existed between tree ring isotopes, iWUE and environmental data at each site (Fig. 2); (2) there was a relationship between iWUE and Δ^18^O_w_ and parameters describing stand development (diameter at the breast height and height, Supplementary Table S2 text S1); (3) changes in iWUE and δ^15^N_w_ were correlated with age and soil type (Supplementary text S2 and S3). Subsequently, we considered models jointly allowing explanation of spatial and temporal variation in isotopic data. To explain temporal trends in isotopic data (iWUE, Δ^18^O_w_, c_i_/c_a,_ δ^15^N_w_) jointly across multiple sites, we considered both explanatory variables that varied yearly for each site and mean climatic data averaged over time for each site. Yearly time series and mean climatic data for each site (mean temperature, maximum temperature, precipitation and vapour pressure deficit VPD) were calculated for each year (from 1980 to 2010) and also separately for each growing season. We considered SPEI for the month of August with a time-scale of one, two and three months and then the SPEI for the month of December with a time-scale of one and twelve months. By definition, being centered around zero, SPEI defines yearly anomalies and cannot be used as a site index. We also included both the annual deposition data (i.e., aS_dep_ and aN_dep_, to assess the effect of temporal changes) and the mean over the monitoring time (i.e., sS_dep_ and sN_dep_) to evaluate both the contribution of within-site temporal changes and cross-sites differences on changes in iWUE, c_i_/c_a_, Δ^18^O_w_ and δ^15^N_w_. Note that we only have data for the aSdep and aNdep at 10 of the 12 sites. To eliminate auto-correlation between mean site variables and yearly variables for each site, site variables were globally centered, whereas yearly data were group-centered^59^. To eliminate auto-correlation among individual climatic variables of the two groups, we conducted two principal components (PC) analyses, after centering and scaling the variables. The first PC analysis considered across sites long-term averages of environmental variables (PCA_s), the second within sites annual time series (PCA_a). Tables of percentage of variance explained and scree plots were examined to determine how many PC to retain. Linear mixed models (using library *nlme* in R^60^) were employed for each of the isotope data series to explain temporal and spatial patterns of variation as a function of climatic and atmospheric deposition conditions. We also ran the linear mixed model without the two Sitka spruce stands, in order to account for other source of variations (e.g., age-effect) for the isotope parameters and iWUE (particularly for the youngest stand at the Goyt site). To explore the significance of systematic differences among the 12 (or 10, when Sitka spruce was excluded) sites occupied by the two evergreen and the two deciduous species, a categorical variable with two levels (combining species into two functional groups) was introduced as a fixed factor. Since multiple species were present at some of the sites, an identification factor for each site × species combination was employed as random factor. The initial models included all PC previously identified as potential explanatory variables for both spatial and temporal variation and also forest stand-related (age and soil type) and anthropogenic (atmospheric CO_2_, N_dep_ and S_dep_) factors (Ref. Supplementary Table S7 including all the models tested and reference to tables and supplementary text where results are reported). These models were then gradually simplified until the minimal significant model was achieved, i.e., excluding all PC and other factors that were not significant following simultaneous tests for general linear hypothesis testing (package *multicomp*,^61^). A correlation structure of order 1 was included in the model for each site × species combination to allow for the temporal dependency of measurements carried out in subsequent years. In the case of nested models, significance was tested using a chi-square test with one degree of freedom. Quality of fit was assessed using residual distribution plots, qqnorm plots of standardised residuals against quantiles of standard normals for both individual points and for the random effects, and auto-correlation function plots of normalized residuals as a function of measurement lags. Marginal (only fixed factors) and conditional (fixed + random factors) percent of explained variance (R^2^_m_ and R^2^_c_, respectively) were calculated using package *MuMIn*^62^.

Finally, to examine the joint effects of climatic conditions on Δ^18^O_w_, δ^15^N_w_ and iWUE, a mixed-effect confirmatory path model was employed using package *piecewiseSEM*^63^. For each isotope and iWUE, the final model from linear mixed effect model analyses (Tables 3, 4, Supplementary Table S7) was considered, with the only modification of excluding PFT as fixed factor and including also Δ^18^O_w_ as fixed factor in the model for iWUE and the correlated errors between PC_s1 and sNdep. *PiecewiseSEM* allows the fitting of hierarchical models with random effects on data with a multivariate structure, allowing for the identification of indirect effects and unresolved covariance among endogenous variables. Model goodness-of-fit was assessed using a chi-square test against Fisher C, based on Shipley’s test of direct separation, which tests for the overall significance of missing relationships among unconnected variables, while the significance of any given missing path was evaluated using individual *p*-values. A combination of non-significant Fisher-C and individual *p*-values tests implies that the hypothesised relationships among the variables are consistent with the data without missing any significant relationship^63^. All statistical analyses were carried out inside RStudio 1.0.143 with R 3.4.0^64^.

## Supplementary information

Supplementary file1 (PDF 1557 kb)

## Data Availability

All isotope data used in this paper are available on Zenodo: 10.5281/zenodo.3907849.
