# Supplementary material for: Climate and atmospheric deposition effects on forest water-use efficiency and nitrogen availability across Britain
Source: Sci Rep. 2020 Jul 24;10:12418. doi: 10.1038/s41598-020-67562-w (PMC7381603; doi:10.1038/s41598-020-67562-w)
Supplement: Supplementary file 1 — Supplementary file1 (PDF 1557 kb) [file 41598_2020_67562_MOESM1_ESM.pdf]

Supplementary information for:

Climate and atmospheric deposition effects on forest water-use efficiency and nitrogen availability across Britain

Guerrieri Rossella, Vanguelova Elena, Pitman Rona, Benham Sue, Perks Mike, Morison James, Mencuccini Maurizio

Corresponding author:
Rossella Guerrieri
Email: rossellaguerrieri@gmail.com

The PDF file includes:
Tables S1 to S7
Figure S1 to S5
Supplementary text: S1 Output from linear regression analyses to assess relationships between iWUE or $\Delta^{18}O_w$ *vs.* change in tree height, Diameter at the breast height and basal area; S2-S3 Output from linear regression analyses to assess relationships between iWUE or $\delta^{15}N_w$ *vs.* age and soil types; S4-S6: Output from Linear mixed model analyses for iWUE, $\Delta^{18}O_w$ and $\delta^{15}N_w$ including or not Sitka spruce stands (starting from full models with all parameters included and all steps leading to the final models shown in the tables 3 and 4 in the main text).

Table S1. **Mean atmospheric deposition at the investigated sites.** The mean of total deposition (NH_4-N + NO_3-N) as calculated for the years 1995-2010 at most of the sites, with the exception of Rogate, where we only had one year of monitoring. For Shobdon and Covert Wood, which were not part of the regular ICP forest water chemistry-monitoring network, we considered deposition data as obtained from the UK 5x5 km grid N_{dep} and S_{dep} dataset from RoTAP, (2012) (see methods for details).

**Site**	**Species**	**Year monitoring**	**Mean Total deposition (kg ha^{-1} yr^{-1})**	
			**N_{dep}**	**S_{dep}**
Alice Holt	Fagus sylvatica		9.8	9.6
	Quercus robur	1995-2010		
Covert Wood	Fagus sylvatica	N_{dep} map	6.7	3.7
Goyt	Picea sitchensis	1995-2010	15.2	16.5
LadyBower	Pinus sylvestris			
Rannoch	Pinus sylvestris	1995-2010	4.8	5.1
Rogate	Pinus sylvestris	2010	10.7	4.1
Savernake	Quercus robur	1995-2010	12.7	10.9
Shobdon	Fagus sylvatica	N_{dep} map	14.8	6.6
Thetford	Fagus sylvatica	1995-2010	19.7	9.5
	Pinus sylvestris	1995-2010	13.6	7.5
Tummel	Picea sitchensis	1995-2010	4.8	6.4

Table S2. **Temporal changes in tree iWUE across the investigated sites.** Relative changes in iWUE (i.e., value at 2010 minus value at 1980 divided by value at 1980, iWUE (%)) and slope (μmol mol^{-1} $year^{-1}$) ± standard error (SE) from linear regression analyses exploring i) trends in iWUE over the investigated years and ii) relationship between iWUE and $\Delta^{18}Ow$ for the four species at the twelve forest stands included in the study. Slopes significantly different from zero were indicated by stars, according to the *p*- values: *, ¥, $p\leq 0.10$, $p\leq 0.05$; **, $p\leq 0.01$; ***, $p\leq 0.001$

**Species**	**Site**	**iWUE (%)**	**iWUE *vs.* year**	**iWUE *vs.* $\Delta^{18}O_w$**
			Slope ±SE	Slope ±SE
Beech	Shobdon	5.1	0.02 ± 0.18	4.85±2.63¥
	Alice Holt	16.6	0.43 ± 0.14**	1.25±3.63
	Thetford	19.9	0.03 ± 0.18	3.57±2.97
	Covert Wood	19.6	0.14 ± 0.15	3.30±1.94¥
Scots pine	Rannoch	25.8	0.54 ± 0.09***	-4.10±3.14
	Ladybower	17.5	0.36 ± 0.08***	6.03±2.30*
	Thetford	13.6	0.44 ± 0.11***	5.21±1.45**
	Rogate	14.4	0.36 ± 0.11**	1.17±2.59
Oak	Alice Holt	30.5	0.73 ± 0.09***	1.50±3.47
	Savernake	14.5	0.21 ± 0.08*	1.29±1.77
Sitka spruce	Tummel	-15.5	-0.24 ± 0.12¥	7.50±2.58**
	Goyt	-29.6	-1.36 ± 0.12***	-7.55±6.58

Table S3. Differences between tree species at high vs. low N_{dep} sites for tree ring $\delta^{15}N_w$ values. The average of the year-by-year differences and the relative confidence interval are reported.

**Species**	**Site**	**Diff $\delta^{15}N_w$ (‰)**	**CI**
Beech	Thetford vs. Alice Holt	1.57	0.48
Scots pine	Ladybower vs. Rannoch	-1.32	0.41
	Thetford vs. Rogate	-0.79	0.60
Oak	Savernake vs. Alice Holt	-1.00	0.33
Sitka spruce	Goyt vs. Tummel	-1.84	0.70

Table S4. Relationship between iWUE *vs.* climate factors and atmospheric CO_2 per level of N_{dep}. Statistics of the linear mixed model analyses for the regression of iWUE as a function of the site (PC_s) and time (PC_a) climate variables from PCA analysis, and changes in atmospheric CO_2 (c_a). Output is given separately for the forest stands at high and low Ndep and with or without the Sitka spruce stands. Estimate of slope and intercept and standard error (SE) values are provided for each of the fixed factor. Marginal (only fixed factors) and conditional (fixed + random factors) proportions of the explained variance are indicated as R^2_m and R^2_c, respectively.

Fixed effects	Estimate ± SE		Auto-correlation structure	Random effects standard deviation	Residual variance	R^2_m	R^2_c
**i) High N_{dep} sites - All sites**			0.56	6.20	5.18	0.15	0.44
Intercept	83.79±4.10	***					
c_a	0.02±0.09	n.s.					
PCA_s1	-3.36±2.61	n.s.					
PCA_a1	-0.46±0.17	**					
PCA_a3	-0.35 ±0.28	n.s.					
**ii) High N_{dep} sites - without Sitka spruce**			0.49	6.63	5.09	0.26	0.72
Intercept	81.82±4.14	***					
c_a	0.13±0.06	*					
PCA_s1	-3.39±2.45	n.s.					
PCA_a1	-0.48 ±0.20	*					
PCA_a3	-0.25±0.31	n.s.					
**iii) Low N_{dep} - All sites**			0.59	4.96	5.29	0.40	0.68
Intercept	83.48±2.38	***					
c_a	0.16±0.06	**					
PCA_s1	-1.67± 0.74	**					
PCA_a1	-0.84±0.19	0.08					
PCA_a3	-0.30±0.29	n.s.					
**iv) Low N_{dep} sites - without Sitka spruce**			0.51	5.32	4.69	0.38	0.73
Intercept	84.14±2.58	***					
c_a	0.23±0.05	***					
PCA_s1	-1.31± 0.87	n.s.					
PCA_a1	-0.81± 0.19	**					
PCA_a3	-0.32±0.30	n.s.					

Table S5. Mean ± standard error (SE) calculated over n=10 trees for the year 2007 for each species at eight of the twelve forest stands for $\delta^{13}C_w$ and $\delta^{18}O_w$.

**Site**	**Species**	**$\delta^{13}C_w$**	**SE**	**$\delta^{18}O_w$**	**SE**
Alice Holt	Beech	-28.18	0.66	25.44	0.24
	Oak	-27.11	0.27	24.67	0.17
Goyt	Sitka spruce	-27.73	0.31	22.69	0.14
Ladybower	Scots pine	-26.66	0.28	23.34	0.30
Rogate	Scots pine	-25.98	0.31	24.76	0.16
Savernake	Oak	-27.53	0.23	24.32	0.18
Thetford	Beech	-28.01	0.80	25.83	0.18
Tummel	Sitka spruce	-28.00	0.16	22.19	0.14

Table S6. Comparing estimates of $\delta^{18}O$ in precipitation ($\delta^{18}O_P$) as obtained from eq. 5 (ref. Methods) with those obtained from the Online Isotopes in Precipitation Calculator,http://wateriso.utah.edu/waterisotopes/pages/data_access/oipc.html) and measured values at Keyworth in England. We report i) the mean of estimated $\boldsymbol{\delta^{18}O_P}$ values over the years considered in this study (1980-2010), ii) single value as obtained from the online calculator, by including coordinates and elevation [*]; iii) mean of measured $\delta^{18}O_P$ values from 1985 to 1996 [#]. Mean ± standard deviation values across all sites are -8.2±0.5 and -7.5±0.6 for estimates obtained in this study and those from Bowen et al. 2020, respectively.

**Species**	**Site**	**Estimated $\delta^{18}O_P$**		**Measured $\delta^{18}O_P$**
		This study	Bowen (2020)	IAEA
Beech	Shobdon	-8.1	-7.8	-7.7 ±0.7
	Alice Holt	-7.9	-7.1	-7.7 ±0.7
	Thetford	-7.8	-7.5	-7.7 ±0.7
	Covert Wood	-7.5	-6.9	-7.7 ±0.7
Scots pine	Rannoch	-9.1	-8.1	-7.7 ±0.7
	Ladybower	-8.6	-8.4	-7.7 ±0.7
	Thetford	-7.8	-7.5	-7.7 ±0.7
	Rogate	-7.9	-7	-7.7 ±0.7
Oak	Alice Holt	-7.9	-7.1	-7.7 ±0.7
	Savernake	-7.9	-7.3	-7.7 ±0.7
Sitka spruce	Tummel	-9.0	-7.0	-7.7 ±0.7
	Goyt	-8.6	-8.6	-7.7 ±0.7

[*] Bowen, G. J. (2020) The Online Isotopes in Precipitation Calculator, version OIPC3.1 (4/2017). http://www.waterisotopes.org. Bowen G. J. and Revenaugh J. (2003). Interpolating the isotopic composition of modern meteoric precipitation. *Water Resources Research* **39** (10), 1299.

[#] IAEA/WMO (2015). Global Network of Isotopes in Precipitation. The GNIP Database. Accessible at: https://nucleus.iaea.org/wiser.

Table S7. Summary of all statistical models tested in this study and reference (Ref.) to the figure/table or supplementary text where results are reported. LM, LME and SEM indicate linear, linear mixed-effect and structural equation models.

N of sites	Analysis - Equations	Ref.
Linear regression		
All	iWUE~Year	Fig. 2, Table S2
	c_i/c_a ~Year	Fig. 2
	$\Delta^{18}O_w$~Year	Fig. 2
	$\delta^{15}N_w$~Year	Fig. 2
	iWUE ~ tree height; diameter at the breast height; basal area<br>$\Delta^{18}O_w$ ~ tree height; diameter at the breast height; basal area	Text S1
Linear mixed models (LME) – iWUE all sites		
12	iWUE ~ Age + Soil type	Text S2
10	iWUE ~ PFT + pca_s1 + pca_a1 + pca_a3 + sNdep + sSdep + c_a + aSdep + aNdep	Table 3
12	iWUE~ PFT + pca_s1 + pca_a1 + pca_a3 + sSdep +sNdep+ c_a	Text S4
	iWUE ~ PFT + pca_s1 + pca_a1 + pca_a3+ c_a	Text S4
	iWUE ~ PFT + pca_s1 + pca_a1 + pca_a3	Table 3
	iWUE ~ PFT + Age + Soil type + pca_s1 + pca_a1 + pca_a3	Text S2
Linear mixed models (LME) – iWUE without Sitka spruce stands		
8	iWUE ~ PFT + pca_s1 + pca_a1 + pca_a3 + sNdep + sSdep + c_a + aSdep + aNdep	Text S4
	iWUE ~ PFT + pca_s1 + pca_a1 + pca_a3 + sSdep + aSdep	Table 3
10	WUE~ PFT + pca_s1 + pca_a1 + pca_a3 + sSdep +sNdep+ c_a	Text S4
	WUE~ PFT + pca_s1 + pca_a1 + pca_a3 + sSdep + c_a	Table S4
	WUE~ PFT + pca_s1 + pca_a1 + c_a	Table 3
Linear mixed models (LME) – iWUE per level of Ndep, with or without Sitka spruce stands		
6 low<br>6 high	iWUE ~ PFT + pca_s1 + pca_a1 + pca_a3 + c_a	Table S4
Linear mixed models (LME) – $\Delta^{18}O_w$ all sites and all parameters		
10	$\Delta^{18}O_w$ ~ pca_s1 + pca_a1 + pca_a2 + pca_a3 + c_a + PFT + sNdep + sSdep + aNdep+aSdep (all parameters)	Text S5
	$\Delta^{18}O_w$ ~ pca_s1 + pca_a1 + pca_a2 + pca_a3 +PFT + aSedep+ sSdep (final model for all sites model)	Table 4
12	$\Delta^{18}O_w$ ~ pca_s1 + pca_a1 + pca_a2 + pca_a3 +PFT + sSdep+sNdep+c_a (all parameters)	Text S5
	$\Delta^{18}O_w$ ~ pca_s1 + pca_a1 + pca_a2 + pca_a3 +PFT+ sSdep (final model for all parameters model)	Table 4
Linear mixed models (LME) – $\Delta^{18}O_w$ without Sitka spruce stands – all parameters and all sites		
8	$\Delta^{18}O_w$ ~ pca_s1 + pca_a1 + pca_a2+ pca_a3 + c_a + PFT + sNdep + sSdep + aNdep+aSdep (all parameters)	Text S5
10	$\Delta^{18}O_w$ ~ pca_s1 + pca_a1 + pca_a3 + PFT + cNdep + cSdep+ c_a (best model for all parameters = model all sites)	Text S5
	$\Delta^{18}O_w$ ~ pca_s1 + pca_a1 + pca_a2 + PFT (final model for all parameters and all sites)	Table 4
Linear mixed models (LME) – $\delta^{15}N_w$ all sites		
10	$\delta^{15}N_w$ ~ PFT + pca_s1 + pca_a1 + pca_a3 + c_a+ sNdep + aNdep	Text S6

	$\delta^{15}N_w$ ~ PFT + pca_s1 + pca_a1 + pca_a3 + sNdep + aNdep	Text S6
	$\delta^{15}N_w$ ~ PFT + pca_s1 + Ndep_lev	Text S6
	$\delta^{15}N_w$ ~ Years * Ndep_lev	Text S6
	$\delta^{15}N_w$ ~ PFT + pca_s1 + sNdep	Table 4
	$\delta^{15}N_w$ ~ +pca_s1 + sNdep + Age + Soil type	Text S3
Linear mixed models (LME) – $\delta^{15}N_w$ without Sitka spruce stands		
8	$\delta^{15}N_w$ ~ PFT + pca_s1 + pca_a1 + pca_a3 + c_a+ sNdep + aNdep	Text S6
	$\delta^{15}N_w$ ~ PFT + pca_s1 + Ndep_lev	Text S6
	$\delta^{15}N_w$ ~ Years * Ndep_lev	Text S6
	$\delta^{15}N_w$ ~ PFT + pca_s1 + sNdep	Text S6
SEM – all sites (We considered final equations from LME models, but PFT was excluded and $\Delta^{18}O_w$ was included in the iWUE model)		
12	lme(iWUE ~ pca_a1 + pca_s1 + pca_a3 + $\Delta^{18}O_w$, correlation=corAR1(0, form=~1|Site_Sp), random=~1|Site_Sp)<br>lme($\Delta^{18}O_w$ ~ pca_s1 + pca_a1 + pca_a2+ pca_a3+sSdep , correlation=corAR1(0, form=~1|Site_Sp), random=~1|Site_Sp)<br>lme($\delta^{15}N_w$ ~ pca_s1 + sNdep, correlation=corAR1(0, form=~1|Site_Sp), random=~1|Site_Sp)<br>pca_s1%~~% sNdep (to account for covariance between pca_s1 and sNdep)	Fig.4A
SEM – all sites without Sitka spruce (We considered final equations from LME models, but PFT was excluded and $\Delta^{18}O_w$ was included in the iWUE model)		
10	lme(iWUE ~ pca_a1 + pca_s1 +$\Delta^{18}O_w$ +c_a, correlation=corAR1(0, form=~1|Site_Sp), random=~1|Site_Sp)<br>lme($\Delta^{18}O_w$ ~ pca_a1 + pca_a2, correlation=corAR1(0, form=~1|Site_Sp), random=~1|Site_Sp)<br>lme($\delta^{15}N_w$ ~ pca_s1 + sNdep, correlation=corAR1(0, form=~1|Site_Sp), =~1|Site_Sp)<br>pca_s1 %~~% sNdep	Fig. 4B
SEM – all parameters without Sitka spruce (We considered final equations from LME models, but PFT was excluded and $\Delta^{18}O_w$ was included in the iWUE model)		
8	lme(iWUE ~ pca_a1 + pca_s1 + pca_a3 + $\Delta^{18}O_w$ + sSdep + aSdep, correlation=corAR1(0, form=~1|Site_Sp), random=~1|Site_Sp)<br>lme($\Delta^{18}O_w$ ~ pca_s1 + pca_a2, correlation=corAR1(0, form=~1|Site_Sp), random=~1|Site_Sp)<br>lme($\delta^{15}N_w$ ~ pca_s1 + sNdep, correlation=corAR1(0, form=~1|Site_Sp), random=~1|Site_Sp)<br>pca_s1 %~~% sNdep	Fig. S4

Figure S1. Results from the PCA analyses. Scree plots of the PCA analyses including across sites long-term averages of environmental variables (PCA_s, panel A) and within sites annual time series (PCA_a, panel 2). Next to each plot we also report the tables of percentage of variance explained by the retained PC axis and considered in the statistical analyses reported in the main text.

A)

Site PCA analysis (PCA_s)

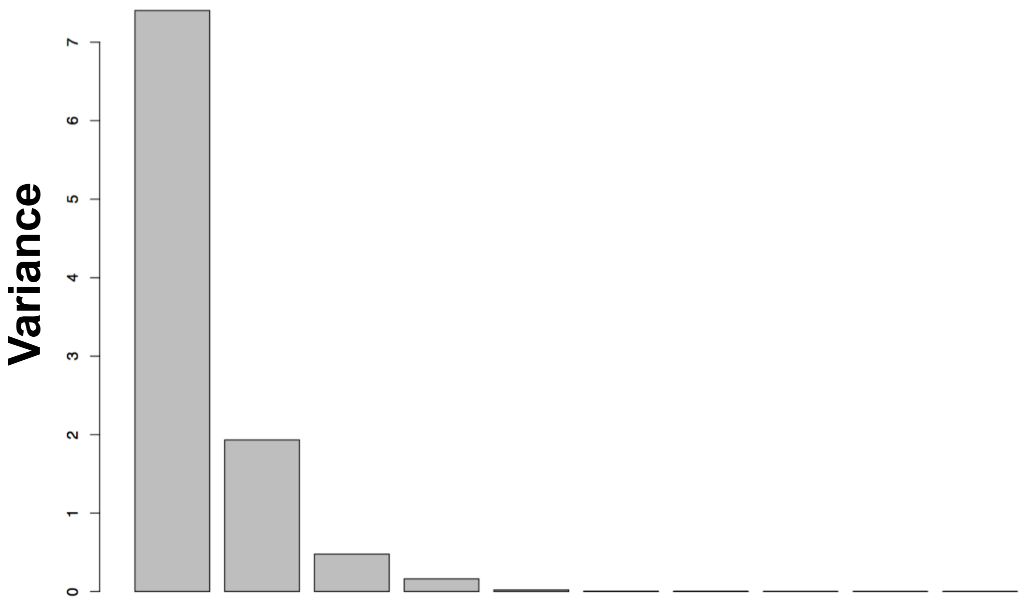

	PC1	PC2
Prec_grs	0.3429950	-0.169030097
Prec_annual	0.3595289	0.006232595
VPD_grs	-0.3298303	-0.550739278
VPD_annual	-0.3007576	-0.665895925
T_grs	-0.3729435	0.275024023
T_annaul	-0.3586107	0.327860946
Tmax_grs	-0.3768732	0.106149050
Tmax_annual	-0.3796423	0.173954628

B)

Annual PCA analysis (PC_a)

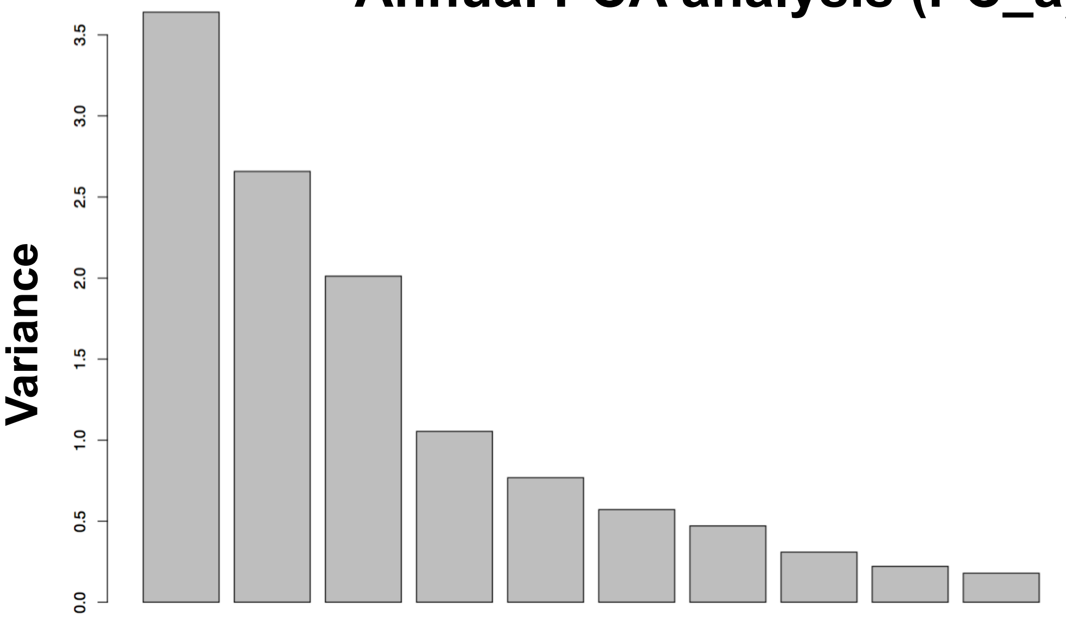

	PC1	PC2	PC3
SPEI8_1	0.19236594	0.06015290	-0.53304052
SPEI8_2	0.28107766	0.04652127	-0.49601301
SPEI12_1	-0.01250284	-0.39401102	0.26130785
SPEI12_12	0.35555742	-0.29464376	-0.09286985
Tmax_grs	-0.34421189	-0.20628203	-0.23548662
T_grs	-0.27939844	-0.32416604	-0.25092423
Tmax_annual	-0.26450909	-0.40729600	-0.02815323
T_annual	-0.16441678	-0.49641964	0.01189985
P_grs	0.32276232	-0.21031195	-0.20731192
P_annual	0.31097367	-0.32217869	-0.14714354
VPD_grs	-0.37039635	0.15812636	-0.31827907
VPD_annual	-0.35467795	0.13843849	-0.32810082

Figure S2. Tree-ring δ^{15}N *vs.* %N. Linear regression analyses to assess relationships between δ^{15}N and %N values measured in tree rings. Only sites where relationships were significant are reported (i.e., AH=Alice Holt; TH=Thetford; RO=Rogate;

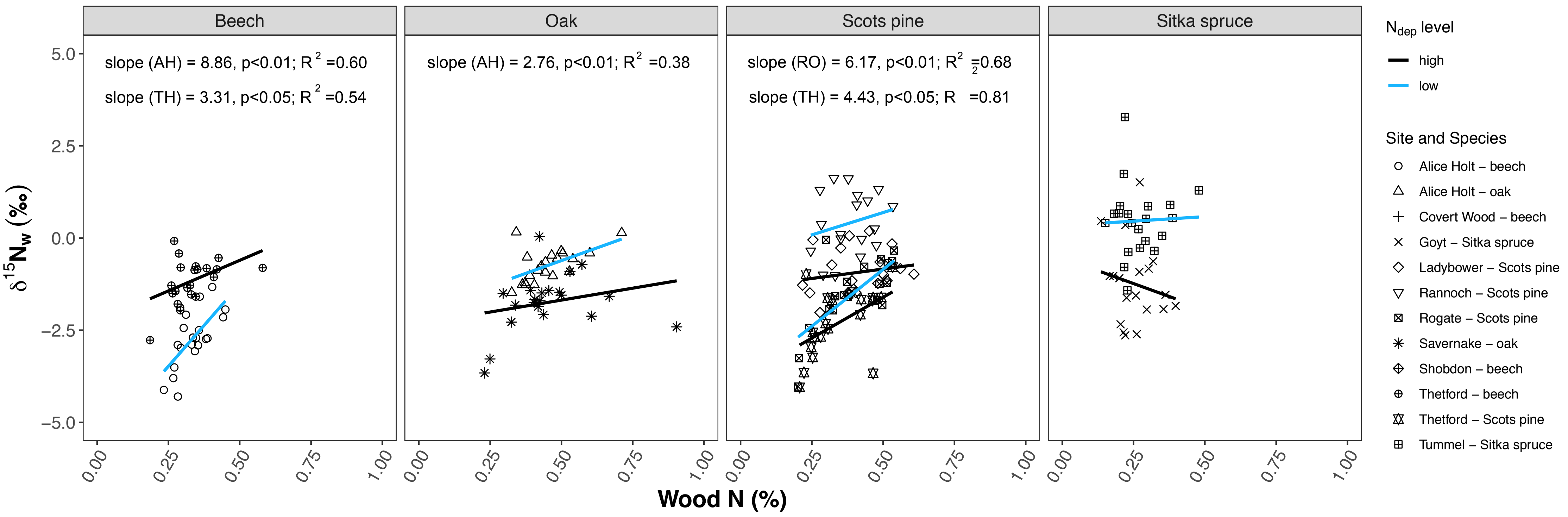

Beech
Oak
Scots pine
Sitka spruce
slope (AH) = 8.86, p<0.01; R2 =0.60
slope (TH) = 3.31, p<0.05; R2 =0.54
slope (AH) = 2.76, p<0.01; R2 =0.38
slope (RO) = 6.17, p<0.01; R2 =0.68
slope (TH) = 4.43, p<0.05; R2 =0.81
δ15Nw (‰)
Wood N (%)
Ndep level
high
low
Site and Species
Alice Holt – beech
Alice Holt – oak
Covert Wood – beech
Goyt – Sitka spruce
Ladybower – Scots pine
Rannoch – Scots pine
Rogate – Scots pine
Savernake – oak
Shobdon – beech
Thetford – beech
Thetford – Scots pine
Tummel – Sitka spruce

Figure S3. Comparison of iWUE and $\Delta^{18}O_w$ for two species at the same site. Boxplot showing iWUE and $\Delta^{18}O_w$ values measured for oak and beech trees at Alice Holt.

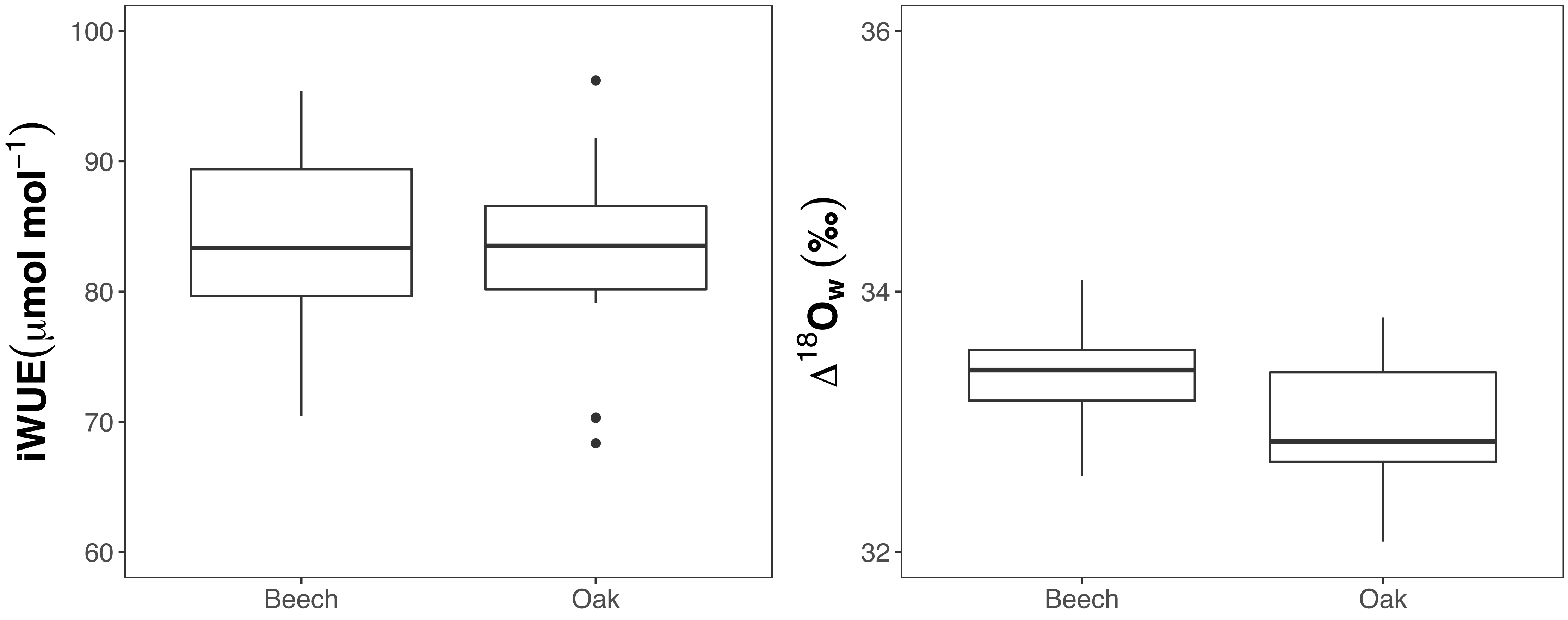

iWUE(μmol mol⁻¹)
100
90
80
70
60
Beech
Oak
Δ18Ow (‰)
36
34
32
Beech
Oak

Figure S4. Stand-related parameters at seven of the twelve investigated sites. Boxplots showing variations in mean tree height (m), diameter and breast height (DBH, cm) and basal area (m^2/ha). Full names of sites and species: AHOA= Alice Holt, oak (5); SAOA= Savernake, oak (4); COBE= Covert Wood, beech (3); THSP= Thetford, Scots pine (4); LBSP = Ladybower, Scots pine (5); RASP= Rannoch, Scots pine (4); TUSS= Tummel, Sitka spruce (5). In brackets are indicated the number of years data were available for. Note that Cover Wood was removed from the analyses shown in the Supplementary text S1, as for some parameters there were data only for 2 years.

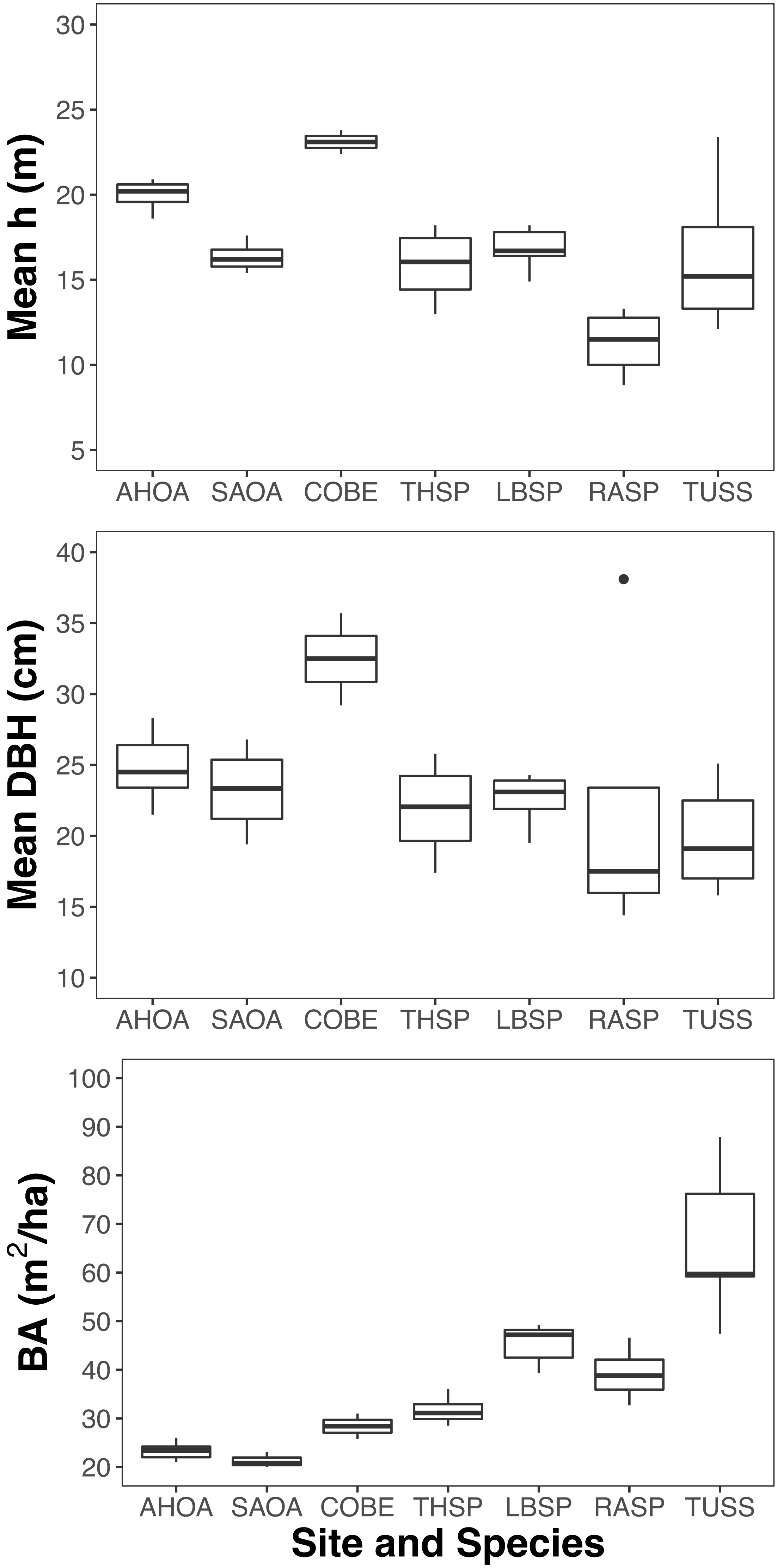
Mean h (m)
30
25
20
15
10
5
AHOA
SAOA
COBE
THSP
LBSP
RASP
TUSS
Mean DBH (cm)
40
35
30
25
20
15
10
AHOA
SAOA
COBE
THSP
LBSP
RASP
TUSS
BA (m2/ha)
100
90
80
70
60
50
40
30
20
AHOA
SAOA
COBE
THSP
LBSP
RASP
TUSS
Site and Species

Figure S5. Direct and indirect effects on tree-ring stable isotopes and derived physiological parameters. Result from the structural equation modelling analysis, which included all parameters and all sites but the two Sitka spruce stands. Continuous arrows indicate relationships significant at the $p \leq 0.05$ or greater (depending on number of stars, i.e., *, $p \leq 0.05$; **, $p \leq 0.01$; ***, $p \leq 0.001$). Dashed arrows (and the '¥' symbol) indicate relationship significant at $p \leq 0.10$. Thickness of lines reflects level of significance. Black and blue arrows indicate negative and positive relationships, respectively. Double-headed arrow indicates correlated errors between variables. PC_s1 indicates the first component from the PCA_s (using long-term average climate variables across sites) and PC_a1 and PC_a3 the first two principal components from the PCA_a (using annual climatic anomalies). Numbers next to the paths indicate standardized path coefficients. Numbers below each isotope-related parameters indicate marginal and conditional R^2, respectively as obtained from LME models described in the Table S7. Notice that they are not the same as reported in Tables 3 and 4 (i.e., PFT was not included as fixed factor in any model, while $\Delta^{18}O_W$ was included as fixed factor in the model for iWUE).

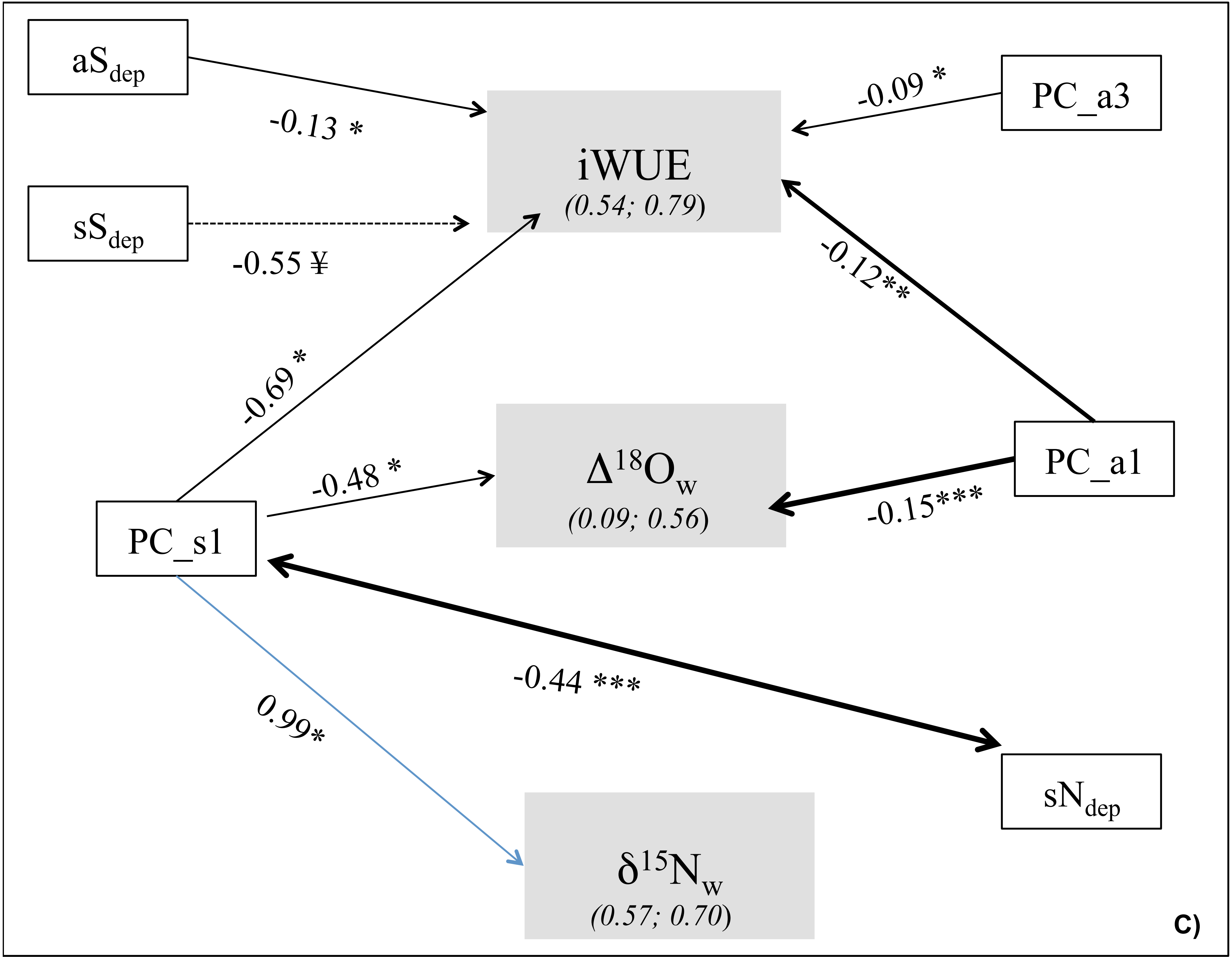

aS$_{dep}$
-0.13 *
sS$_{dep}$
-0.55 ¥
iWUE
(0.54; 0.79)
-0.09 *
PC_a3
-0.12**
-0.69 *
$\Delta^{18}O_w$
(0.09; 0.56)
PC_a1
-0.48 *
-0.15***
PC_s1
-0.44 ***
0.99*
sN$_{dep}$
$\delta^{15}N_w$
(0.57; 0.70)
C)

```
##################################################################################
############################Supplementary text S1##################################
##################################################################################
##################################################################################
Output from linear regression between iWUE or Oxygen isotope discrimination (D18Ow) and
stand parameters measured 4-5 years within the 1995-2010 time-window.

For full name of sites and Species refer to Caption to Figure S4.
##################################################################################
SiteSP= Site and Species
##################################################################################


###Relationship between iWUE and mean diameter at the breast height (Mean DBH) for each
forest stand###

   SiteSP (Intercept)    Mean DBH
1    AHOA    69.24055  0.6939927
2    LBSP    41.24976  1.7220454
3    RASP    82.98656 -0.2853592
4    SAOA    75.94862  0.3908009
5    THSP    82.00124  1.0572559
6    TUSS    75.43735 -0.1640974

Call:
lm(formula = iWUE ~ Mean DBH, data = data)

Residuals:
      1       2       3       4       5 
 3.3673 -0.5380 -4.9604 -0.7428  2.8740 

Coefficients:
            Estimate Std. Error t value Pr(>|t|)  
(Intercept)  69.2406    18.3400   3.775   0.0325 *
Mean DBH      0.6940     0.7356   0.943   0.4151  
---
Signif. codes:  0 ‘***’ 0.001 ‘**’ 0.01 ‘*’ 0.05 ‘.’ 0.1 ‘ ’ 1

Residual standard error: 3.875 on 3 degrees of freedom
Multiple R-squared:  0.2288,    Adjusted R-squared:  -0.02827 
F-statistic:  0.89 on 1 and 3 DF,  p-value: 0.4151


Call:
lm(formula = iWUE ~ Mean DBH, data = data)

Residuals:
     1      2      3      4      5 
 1.702 -3.805  1.234 -1.294  2.164 

Coefficients:
            Estimate Std. Error t value Pr(>|t|)  
(Intercept)  41.2498    16.9833   2.429   0.0934 .
Mean DBH      1.7220     0.7513   2.292   0.1057  
---
Signif. codes:  0 ‘***’ 0.001 ‘**’ 0.01 ‘*’ 0.05 ‘.’ 0.1 ‘ ’ 1

Residual standard error: 2.901 on 3 degrees of freedom
Multiple R-squared:  0.6365,    Adjusted R-squared:  0.5154 
F-statistic: 5.254 on 1 and 3 DF,  p-value: 0.1057


Call:
lm(formula = iWUE ~ Mean DBH, data = data)

Residuals:
      1       2       3       4 
-0.3853 -1.3454 -1.0179  2.7486 

Coefficients:
            Estimate Std. Error t value Pr(>|t|)   
(Intercept)  82.9866     2.8882  28.733  0.00121 **
```

```
Mean DBH      -0.2854     0.1211  -2.355  0.14265
---
Signif. codes:  0 ‘***’ 0.001 ‘**’ 0.01 ‘*’ 0.05 ‘.’ 0.1 ‘ ’ 1

Residual standard error: 2.297 on 2 degrees of freedom
Multiple R-squared:  0.735,     Adjusted R-squared:  0.6026
F-statistic: 5.548 on 1 and 2 DF,  p-value: 0.1427


Call:
lm(formula = iWUE ~ Mean DBH, data = data)

Residuals:
      1       2       3       4
-0.1776  1.7669 -3.9579  2.3686

Coefficients:
            Estimate Std. Error t value Pr(>|t|)
(Intercept)  75.9486    14.4000   5.274   0.0341 *
Mean DBH       0.3908     0.6154   0.635   0.5904
---
Signif. codes:  0 ‘***’ 0.001 ‘**’ 0.01 ‘*’ 0.05 ‘.’ 0.1 ‘ ’ 1

Residual standard error: 3.495 on 2 degrees of freedom
Multiple R-squared:  0.1678,    Adjusted R-squared:  -0.2483
F-statistic: 0.4032 on 1 and 2 DF,  p-value: 0.5904


Call:
lm(formula = iWUE ~ Mean DBH, data = data)

Residuals:
      1       2       3       4
 4.1562 -6.1203 -0.8866  2.8508

Coefficients:
            Estimate Std. Error t value Pr(>|t|)
(Intercept)  82.0012    19.4504   4.216   0.0519 .
Mean DBH       1.0573     0.8818   1.199   0.3533
---
Signif. codes:  0 ‘***’ 0.001 ‘**’ 0.01 ‘*’ 0.05 ‘.’ 0.1 ‘ ’ 1

Residual standard error: 5.641 on 2 degrees of freedom
Multiple R-squared:  0.4182,    Adjusted R-squared:  0.1273
F-statistic: 1.438 on 1 and 2 DF,  p-value: 0.3533


Call:
lm(formula = iWUE ~ Mean DBH, data = data)

Residuals:
      1       2       3       4       5
 5.5780 -3.6947 -3.8208  0.3754  1.5620

Coefficients:
            Estimate Std. Error t value Pr(>|t|)
(Intercept)  75.4374    11.8821   6.349  0.00791 **
Mean DBH      -0.1641     0.5883  -0.279  0.79842
---
Signif. codes:  0 ‘***’ 0.001 ‘**’ 0.01 ‘*’ 0.05 ‘.’ 0.1 ‘ ’ 1

Residual standard error: 4.544 on 3 degrees of freedom
Multiple R-squared:  0.02528,   Adjusted R-squared:  -0.2996
F-statistic: 0.07781 on 1 and 3 DF,  p-value: 0.7984


###Relationship between iWUE and mean height (MeanH) for each forest stand###

   SiteSP (Intercept)       MeanH
1    AHOA    63.14791  1.23219888
2    LBSP    38.24942  2.48900210
3    RASP    59.12230  1.56292873
```

```
4    SAOA    53.07875  1.95389708
5    THSP    79.17519  1.63669254
6    TUSS    73.70414 -0.09332065

Call:
lm(formula = iWUE ~ MeanH, data = data)

Residuals:
     1      2      4      5 
 1.462 -2.727 -1.589  2.854 

Coefficients:
            Estimate Std. Error t value Pr(>|t|)
(Intercept)   63.148     36.555   1.727    0.226
MeanH          1.232      1.828   0.674    0.570

Residual standard error: 3.181 on 2 degrees of freedom
  (1 observation deleted due to missingness)
Multiple R-squared:  0.1851,    Adjusted R-squared:  -0.2224 
F-statistic: 0.4542 on 1 and 2 DF,  p-value: 0.5698


Call:
lm(formula = iWUE ~ MeanH, data = data)

Residuals:
     1      2      3      4      5 
 1.196 -3.911  2.447 -1.441  1.710 

Coefficients:
            Estimate Std. Error t value Pr(>|t|)
(Intercept)   38.249     19.716   1.940    0.148
MeanH          2.489      1.171   2.126    0.123

Residual standard error: 3.039 on 3 degrees of freedom
Multiple R-squared:  0.6011,    Adjusted R-squared:  0.4681 
F-statistic:  4.52 on 1 and 3 DF,  p-value: 0.1235


Call:
lm(formula = iWUE ~ MeanH, data = data)

Residuals:
      1       2       3       4 
-1.1470  2.1552 -1.5549  0.5468 

Coefficients:
            Estimate Std. Error t value Pr(>|t|)  
(Intercept)  59.1223     6.6597   8.878   0.0125 *
MeanH         1.5629     0.5834   2.679   0.1157  
---
Signif. codes:  0 ‘***’ 0.001 ‘**’ 0.01 ‘*’ 0.05 ‘.’ 0.1 ‘ ’ 1

Residual standard error: 2.083 on 2 degrees of freedom
Multiple R-squared:  0.7821,    Adjusted R-squared:  0.6731 
F-statistic: 7.177 on 1 and 2 DF,  p-value: 0.1157


Call:
lm(formula = iWUE ~ MeanH, data = data)

Residuals:
      1       2       3       4 
 0.1837  2.0893 -3.5963  1.3233 

Coefficients:
            Estimate Std. Error t value Pr(>|t|)
(Intercept)   53.079     30.832   1.722    0.227
MeanH          1.954      1.883   1.037    0.409

Residual standard error: 3.089 on 2 degrees of freedom
Multiple R-squared:  0.3499,    Adjusted R-squared:  0.02479 
```

```
F-statistic: 1.076 on 1 and 2 DF,  p-value: 0.4085


Call:
lm(formula = iWUE ~ MeanH, data = data)

Residuals:
     1      2      3      4 
 4.101 -6.113 -1.155  3.166 

Coefficients:
            Estimate Std. Error t value Pr(>|t|)  
(Intercept)   79.175     22.576   3.507   0.0726 .
MeanH          1.637      1.415   1.157   0.3669  
---
Signif. codes:  0 ‘***’ 0.001 ‘**’ 0.01 ‘*’ 0.05 ‘.’ 0.1 ‘ ’ 1

Residual standard error: 5.725 on 2 degrees of freedom
Multiple R-squared:  0.4008,    Adjusted R-squared:  0.1012 
F-statistic: 1.338 on 1 and 2 DF,  p-value: 0.3669


Call:
lm(formula = iWUE ~ MeanH, data = data)

Residuals:
      1       2       3       4       5 
 5.8476 -3.5099 -3.8033  0.1056  1.3601 

Coefficients:
            Estimate Std. Error t value Pr(>|t|)   
(Intercept) 73.70414    8.57593   8.594  0.00331 **
MeanH       -0.09332    0.50719  -0.184  0.86575   
---
Signif. codes:  0 ‘***’ 0.001 ‘**’ 0.01 ‘*’ 0.05 ‘.’ 0.1 ‘ ’ 1

Residual standard error: 4.577 on 3 degrees of freedom
Multiple R-squared:  0.01116,   Adjusted R-squared:  -0.3185 
F-statistic: 0.03385 on 1 and 3 DF,  p-value: 0.8658



###Relationship between iWUE and basal area (BA) for each forest stand###
    SiteSP (Intercept)       BA
1    AHOA    89.66025 -0.13699832
2    LBSP    40.30052  0.87818324
3    RASP    54.42948  0.56889335
4    SAOA    44.49318  1.93700312
5    THSP    75.24222  0.94186657
6    TUSS    68.55350  0.05475658

Call:
lm(formula = iWUE ~ BA, data = data)

Residuals:
       1        2        3        4        5 
 0.88236 -1.15633 -5.17155  0.03582  5.40970 

Coefficients:
            Estimate Std. Error t value Pr(>|t|)  
(Intercept)   89.660     26.500   3.383    0.043 *
BA            -0.137      1.133  -0.121    0.911  
---
Signif. codes:  0 ‘***’ 0.001 ‘**’ 0.01 ‘*’ 0.05 ‘.’ 0.1 ‘ ’ 1

Residual standard error: 4.402 on 3 degrees of freedom
Multiple R-squared:  0.004848,  Adjusted R-squared:  -0.3269 
F-statistic: 0.01462 on 1 and 3 DF,  p-value: 0.9114


Call:
lm(formula = iWUE ~ BA, data = data)
```

```
Residuals:
      1       2       3       4       5 
 1.7183 -2.4654  0.5118 -1.5166  1.7519 

Coefficients:
            Estimate Std. Error t value Pr(>|t|)  
(Intercept)  40.3005    11.9154   3.382   0.0430 *
BA            0.8782     0.2622   3.349   0.0441 *
---
Signif. codes:  0 ‘***’ 0.001 ‘**’ 0.01 ‘*’ 0.05 ‘.’ 0.1 ‘ ’ 1

Residual standard error: 2.211 on 3 degrees of freedom
Multiple R-squared:  0.7889,    Adjusted R-squared:  0.7186 
F-statistic: 11.21 on 1 and 3 DF,  p-value: 0.0441


Call:
lm(formula = iWUE ~ Basal, data = data)

Residuals:
      1       2       3       4 
-1.3032  2.0534 -0.2663 -0.4839 

Coefficients:
            Estimate Std. Error t value Pr(>|t|)  
(Intercept)  54.4295     6.8463   7.950   0.0155 *
Basal         0.5689     0.1731   3.287   0.0814 .
---
Signif. codes:  0 ‘***’ 0.001 ‘**’ 0.01 ‘*’ 0.05 ‘.’ 0.1 ‘ ’ 1

Residual standard error: 1.764 on 2 degrees of freedom
Multiple R-squared:  0.8438,    Adjusted R-squared:  0.7657 
F-statistic:  10.8 on 1 and 2 DF,  p-value: 0.08142


Call:
lm(formula = iWUE ~ BA, data = data)

Residuals:
      1       2       3       4 
 0.1193  1.4521 -1.1241 -0.4473 

Coefficients:
            Estimate Std. Error t value Pr(>|t|)  
(Intercept)   44.493     10.714   4.153   0.0534 .
BA             1.937      0.511   3.791   0.0631 .
---
Signif. codes:  0 ‘***’ 0.001 ‘**’ 0.01 ‘*’ 0.05 ‘.’ 0.1 ‘ ’ 1

Residual standard error: 1.339 on 2 degrees of freedom
Multiple R-squared:  0.8778,    Adjusted R-squared:  0.8167 
F-statistic: 14.37 on 1 and 2 DF,  p-value: 0.06308


Call:
lm(formula = iWUE ~ BA, data = data)

Residuals:
     1      2      3      4 
 2.468 -7.839  2.391  2.980 

Coefficients:
            Estimate Std. Error t value Pr(>|t|)
(Intercept)  75.2422    36.7585   2.047    0.177
BA            0.9419     1.1561   0.815    0.501

Residual standard error: 6.408 on 2 degrees of freedom
Multiple R-squared:  0.2492,    Adjusted R-squared:  -0.1262 
F-statistic: 0.6638 on 1 and 2 DF,  p-value: 0.5008
```

```
Call:
lm(formula = iWUE ~ BA, data = data)

Residuals:
      1       2       3       4       5 
 6.6275 -2.1959 -3.3401 -0.6054 -0.4861 

Coefficients:
            Estimate Std. Error t value Pr(>|t|)   
(Intercept) 68.55350    9.52654   7.196  0.00553 **
BA           0.05476    0.14093   0.389  0.72355   
---
Signif. codes:  0 ‘***’ 0.001 ‘**’ 0.01 ‘*’ 0.05 ‘.’ 0.1 ‘ ’ 1

Residual standard error: 4.491 on 3 degrees of freedom
Multiple R-squared:  0.04791,   Adjusted R-squared:  -0.2695 
F-statistic: 0.151 on 1 and 3 DF,  p-value: 0.7235
```

###Relationship between D18Ow and mean diameter at breast Height (Mean DBH) for each forest stand###

```
    SiteSP (Intercept)  Mean DBH
1    AHOA    36.30428 -0.12007985
2    LBSP    30.77911  0.07573824
3    RASP    31.64113  0.02573940
4    SAOA    32.82278  0.02822676
5    THSP    32.02707  0.07334088
6    TUSS    32.01310  0.01545798

Call:
lm(formula = D18O ~ Mean DBH, data = data)

Residuals:
        1         2         3         4         5 
-0.124929  0.005487  0.024312  0.384342 -0.289211 

Coefficients:
            Estimate Std. Error t value Pr(>|t|)    
(Intercept) 36.30428    1.35969  26.700 0.000115 ***
Mean DBH     -0.12008    0.05454  -2.202 0.114977    
---
Signif. codes:  0 ‘***’ 0.001 ‘**’ 0.01 ‘*’ 0.05 ‘.’ 0.1 ‘ ’ 1

Residual standard error: 0.2873 on 3 degrees of freedom
Multiple R-squared:  0.6177,    Adjusted R-squared:  0.4903 
F-statistic: 4.848 on 1 and 3 DF,  p-value: 0.115


Call:
lm(formula = D18O ~ Mean DBH, data = data)

Residuals:
       1        2        3        4        5 
-0.07863  0.11478  0.22420 -0.41776  0.15741 

Coefficients:
            Estimate Std. Error t value Pr(>|t|)    
(Intercept) 30.77911    1.75271  17.561 0.000403 ***
Mean DBH      0.07574    0.07753   0.977 0.400683    
---
Signif. codes:  0 ‘***’ 0.001 ‘**’ 0.01 ‘*’ 0.05 ‘.’ 0.1 ‘ ’ 1

Residual standard error: 0.2994 on 3 degrees of freedom
Multiple R-squared:  0.2413,    Adjusted R-squared:  -0.01157 
F-statistic: 0.9543 on 1 and 3 DF,  p-value: 0.4007


Call:
lm(formula = D18O ~ Mean DBH, data = data)
```

```
Residuals:
       1        2        3        4 
 0.07595  0.40638 -0.08873 -0.39361 

Coefficients:
            Estimate Std. Error t value Pr(>|t|)    
(Intercept) 31.64113    0.51368  61.597 0.000263 ***
Mean DBH     0.02574    0.02155   1.195 0.354704    
---
Signif. codes:  0 ‘***’ 0.001 ‘**’ 0.01 ‘*’ 0.05 ‘.’ 0.1 ‘ ’ 1

Residual standard error: 0.4085 on 2 degrees of freedom
Multiple R-squared:  0.4164,	Adjusted R-squared:  0.1246 
F-statistic: 1.427 on 1 and 2 DF,  p-value: 0.3547


Call:
lm(formula = D18O ~ Mean DBH, data = data)

Residuals:
       1        2        3        4 
 0.31336 -0.61050  0.38613 -0.08899 

Coefficients:
            Estimate Std. Error t value Pr(>|t|)   
(Intercept) 32.82278    2.30870  14.217  0.00491 **
Mean DBH     0.02823    0.09867   0.286  0.80173   
---
Signif. codes:  0 ‘***’ 0.001 ‘**’ 0.01 ‘*’ 0.05 ‘.’ 0.1 ‘ ’ 1

Residual standard error: 0.5603 on 2 degrees of freedom
Multiple R-squared:  0.03931,	Adjusted R-squared:  -0.441 
F-statistic: 0.08184 on 1 and 2 DF,  p-value: 0.8017


Call:
lm(formula = D18O ~ Mean DBH, data = data)

Residuals:
      1       2       3       4 
 0.3743 -0.5263 -0.1437  0.2958 

Coefficients:
            Estimate Std. Error t value Pr(>|t|)   
(Intercept) 32.02707    1.76702  18.125  0.00303 **
Mean DBH     0.07334    0.08011   0.916  0.45656   
---
Signif. codes:  0 ‘***’ 0.001 ‘**’ 0.01 ‘*’ 0.05 ‘.’ 0.1 ‘ ’ 1

Residual standard error: 0.5125 on 2 degrees of freedom
Multiple R-squared:  0.2953,	Adjusted R-squared:  -0.05701 
F-statistic: 0.8382 on 1 and 2 DF,  p-value: 0.4566


Call:
lm(formula = D18O ~ Mean DBH, data = data)

Residuals:
       1        2        3        4        5 
 0.31622  0.06129 -0.46394 -0.25140  0.33783 

Coefficients:
            Estimate Std. Error t value Pr(>|t|)    
(Intercept) 32.01310    1.06359  30.099 8.06e-05 ***
Mean DBH     0.01546    0.05266   0.294    0.788    
---
Signif. codes:  0 ‘***’ 0.001 ‘**’ 0.01 ‘*’ 0.05 ‘.’ 0.1 ‘ ’ 1

Residual standard error: 0.4067 on 3 degrees of freedom
Multiple R-squared:  0.02792,	Adjusted R-squared:  -0.2961 
F-statistic: 0.08617 on 1 and 3 DF,  p-value: 0.7882
```

```
###Relationship between D18Ow and mean height (Mean H) for each forest stand###

    SiteSP (Intercept)    MeanH
1    AHOA    39.51990 -0.31097296
2    LBSP    30.92613  0.09286409
3    RASP    34.57697 -0.21044745
4    SAOA    32.39876  0.06602950
5    THSP    31.85910  0.11176200
6    TUSS    31.91706  0.02458303

Call:
lm(formula = D18O ~ MeanH, data = data)

Residuals:
      1       2       4       5 
-0.1382  0.1684  0.3736 -0.4038 

Coefficients:
            Estimate Std. Error t value Pr(>|t|)  
(Intercept)  39.5199     4.8070   8.221   0.0145 *
MeanH        -0.3110     0.2404  -1.293   0.3251  
---
Signif. codes:  0 ‘***’ 0.001 ‘**’ 0.01 ‘*’ 0.05 ‘.’ 0.1 ‘ ’ 1

Residual standard error: 0.4183 on 2 degrees of freedom
  (1 observation deleted due to missingness)
Multiple R-squared:  0.4555,    Adjusted R-squared:  0.1832 
F-statistic: 1.673 on 1 and 2 DF,  p-value: 0.3251


Call:
lm(formula = D18O ~ MeanH, data = data)

Residuals:
      1       2       3       4       5 
-0.1324  0.1035  0.2759 -0.4076  0.1607 

Coefficients:
            Estimate Std. Error t value Pr(>|t|)    
(Intercept) 30.92613    2.03867  15.170 0.000622 ***
MeanH        0.09286    0.12106   0.767 0.498870    
---
Signif. codes:  0 ‘***’ 0.001 ‘**’ 0.01 ‘*’ 0.05 ‘.’ 0.1 ‘ ’ 1

Residual standard error: 0.3143 on 3 degrees of freedom
Multiple R-squared:  0.164,     Adjusted R-squared:  -0.1147 
F-statistic: 0.5884 on 1 and 3 DF,  p-value: 0.4989


Call:
lm(formula = D18O ~ MeanH, data = data)

Residuals:
       1        2        3        4 
-0.02728  0.02984  0.05177 -0.05432 

Coefficients:
            Estimate Std. Error t value Pr(>|t|)    
(Intercept) 34.57697    0.19271  179.43 3.11e-05 ***
MeanH       -0.21045    0.01688  -12.47  0.00637 ** 
---
Signif. codes:  0 ‘***’ 0.001 ‘**’ 0.01 ‘*’ 0.05 ‘.’ 0.1 ‘ ’ 1

Residual standard error: 0.06027 on 2 degrees of freedom
Multiple R-squared:  0.9873,    Adjusted R-squared:  0.9809 
F-statistic: 155.4 on 1 and 2 DF,  p-value: 0.006373


Call:
```

```
lm(formula = D18O ~ MeanH, data = data)

Residuals:
       1        2        3        4 
 0.26812 -0.62101  0.42350 -0.07061 

Coefficients:
            Estimate Std. Error t value Pr(>|t|)  
(Intercept) 32.39876    5.65461   5.730   0.0291 *
MeanH        0.06603    0.34541   0.191   0.8660  
---
Signif. codes:  0 ‘***’ 0.001 ‘**’ 0.01 ‘*’ 0.05 ‘.’ 0.1 ‘ ’ 1

Residual standard error: 0.5665 on 2 degrees of freedom
Multiple R-squared:  0.01794,   Adjusted R-squared:  -0.4731 
F-statistic: 0.03654 on 1 and 2 DF,  p-value: 0.866


Call:
lm(formula = D18O ~ MeanH, data = data)

Residuals:
      1       2       3       4 
 0.3655 -0.5275 -0.1599  0.3219 

Coefficients:
            Estimate Std. Error t value Pr(>|t|)   
(Intercept)  31.8591     2.0509  15.534  0.00412 **
MeanH         0.1118     0.1286   0.869  0.47630   
---
Signif. codes:  0 ‘***’ 0.001 ‘**’ 0.01 ‘*’ 0.05 ‘.’ 0.1 ‘ ’ 1

Residual standard error: 0.5201 on 2 degrees of freedom
Multiple R-squared:  0.2743,    Adjusted R-squared:  -0.08861 
F-statistic: 0.7558 on 1 and 2 DF,  p-value: 0.4763


Call:
lm(formula = D18O ~ MeanH, data = data)

Residuals:
       1        2        3        4        5 
 0.35904  0.09316 -0.44631 -0.25251  0.24662 

Coefficients:
            Estimate Std. Error t value Pr(>|t|)    
(Intercept) 31.91706    0.73482  43.435 2.69e-05 ***
MeanH        0.02458    0.04346   0.566    0.611    
---
Signif. codes:  0 ‘***’ 0.001 ‘**’ 0.01 ‘*’ 0.05 ‘.’ 0.1 ‘ ’ 1

Residual standard error: 0.3922 on 3 degrees of freedom
Multiple R-squared:  0.09638,   Adjusted R-squared:  -0.2048 
F-statistic:  0.32 on 1 and 3 DF,  p-value: 0.6112



###Relationship between D18Ow and Basal area (BA) for each forest stand###

   SiteSP (Intercept)        Basal
1    AHOA    34.80516 -0.063519156
2    LBSP    30.96025  0.033701432
3    RASP    35.05797 -0.072754424
4    SAOA    35.21602 -0.083043066
5    THSP    31.30962  0.073184288
6    TUSS    31.80146  0.007857909

Call:
lm(formula = D18O ~ BA, data = data)

Residuals:
       1        2        3        4        5 
```

```
 0.18989  0.34623  0.06782  0.04725 -0.65119

Coefficients:
            Estimate Std. Error t value Pr(>|t|)    
(Intercept) 34.80516    2.66260  13.072 0.000967 ***
BA          -0.06352    0.11386  -0.558 0.615861    
---
Signif. codes:  0 ‘***’ 0.001 ‘**’ 0.01 ‘*’ 0.05 ‘.’ 0.1 ‘ ’ 1

Residual standard error: 0.4423 on 3 degrees of freedom
Multiple R-squared:  0.09399,	Adjusted R-squared:  -0.208 
F-statistic: 0.3112 on 1 and 3 DF,  p-value: 0.6159


Call:
lm(formula = D18O ~ BA, data = data)

Residuals:
      1       2       3       4       5 
-0.1073  0.1600  0.2019 -0.4132  0.1586 

Coefficients:
            Estimate Std. Error t value Pr(>|t|)    
(Intercept) 30.96025    1.62810  19.016 0.000318 ***
BA           0.03370    0.03583   0.941 0.416329    
---
Signif. codes:  0 ‘***’ 0.001 ‘**’ 0.01 ‘*’ 0.05 ‘.’ 0.1 ‘ ’ 1

Residual standard error: 0.3021 on 3 degrees of freedom
Multiple R-squared:  0.2277,	Adjusted R-squared:  -0.02971 
F-statistic: 0.8846 on 1 and 3 DF,  p-value: 0.4163


Call:
lm(formula = D18O ~ BA, data = data)

Residuals:
       1        2        3        4 
 0.01885  0.05210 -0.12704  0.05608 

Coefficients:
            Estimate Std. Error t value Pr(>|t|)    
(Intercept) 35.05797    0.41043  85.417 0.000137 ***
BA          -0.07275    0.01038  -7.012 0.019741 *  
---
Signif. codes:  0 ‘***’ 0.001 ‘**’ 0.01 ‘*’ 0.05 ‘.’ 0.1 ‘ ’ 1

Residual standard error: 0.1057 on 2 degrees of freedom
Multiple R-squared:  0.9609,	Adjusted R-squared:  0.9414 
F-statistic: 49.16 on 1 and 2 DF,  p-value: 0.01974


Call:
lm(formula = D18O ~ BA, data = data)

Residuals:
      1       2       3       4 
 0.1286 -0.6611  0.3400  0.1925 

Coefficients:
            Estimate Std. Error t value Pr(>|t|)  
(Intercept) 35.21602    4.40484   7.995   0.0153 *
BA          -0.08304    0.21009  -0.395   0.7308  
---
Signif. codes:  0 ‘***’ 0.001 ‘**’ 0.01 ‘*’ 0.05 ‘.’ 0.1 ‘ ’ 1

Residual standard error: 0.5506 on 2 degrees of freedom
Multiple R-squared:  0.07246,	Adjusted R-squared:  -0.3913 
F-statistic: 0.1562 on 1 and 2 DF,  p-value: 0.7308


Call:
```

```
lm(formula = D18O ~ BA, data = data)

Residuals:
      1       2       3       4 
 0.2821 -0.6473  0.0944  0.2708 

Coefficients:
            Estimate Std. Error t value Pr(>|t|)   
(Intercept) 31.30962    3.09119  10.129  0.00961 **
BA           0.07318    0.09722   0.753  0.53013   
---
Signif. codes:  0 ‘***’ 0.001 ‘**’ 0.01 ‘*’ 0.05 ‘.’ 0.1 ‘ ’ 1

Residual standard error: 0.5389 on 2 degrees of freedom
Multiple R-squared:  0.2208,    Adjusted R-squared:  -0.1688 
F-statistic: 0.5667 on 1 and 2 DF,  p-value: 0.5301


Call:
lm(formula = D18O ~ BA, data = data)

Residuals:
      1       2       3       4       5 
 0.3069  0.1632 -0.4262 -0.2907  0.2467 

Coefficients:
             Estimate Std. Error t value Pr(>|t|)    
(Intercept) 31.801465   0.819622  38.800 3.77e-05 ***
BA           0.007858   0.012125   0.648    0.563    
---
Signif. codes:  0 ‘***’ 0.001 ‘**’ 0.01 ‘*’ 0.05 ‘.’ 0.1 ‘ ’ 1

Residual standard error: 0.3864 on 3 degrees of freedom
Multiple R-squared:  0.1228,    Adjusted R-squared:  -0.1696 
F-statistic:  0.42 on 1 and 3 DF,  p-value: 0.5631


  ##########################################################################################
#############################Supplementary text S2####################################
######################################################################################
######################################################################################
Output from linear mixed model analyses (LME) for iWUE including soil types  and age as
fixed factors. Soil type categories are:arenosol, cambisol, leptosol, podzol, vertisol.
For tree age see table 1 ###############################################
######################################################################################
######################################################################################

###Model I: LME with only stand factors###
###Note that none of them turns out as significant predictor###

Linear mixed-effects model fit by maximum likelihood
 Data: ICP 
       AIC      BIC    logLik
  1558.428 1590.157 -770.2142

Random effects:
 Formula: ~1 | Site_Sp
        (Intercept) Residual
StdDev:    5.425154 7.586495

Correlation Structure: AR(1)
 Formula: ~1 | Site_Sp 
 Parameter estimate(s):
      Phi 
0.7553534 
Fixed effects: iWUE ~ Age + SoilType 
                     Value Std.Error  DF   t-value p-value
(Intercept)      106.25481 11.593161 239  9.165301  0.0000
Age               -0.20421  0.171728   6 -1.189155  0.2793
SoilTypeCambisol -14.79140  8.303224   6 -1.781404  0.1251
SoilTypeleptosol -11.27511  8.285447   6 -1.360832  0.2225
SoilTypepodzol   -13.95455  6.207740   6 -2.247927  0.0656
```

```
SoilTypevertisol  -8.23876  6.414783   6 -1.284340  0.2464
 Correlation: 
                 (Intr) Age    SlTypC SlTypl SlTypp
Age              -0.911                            
SoilTypeCambisol -0.303  0.072                     
SoilTypeleptosol -0.266  0.031  0.335              
SoilTypepodzol   -0.692  0.411  0.473  0.457       
SoilTypevertisol -0.059 -0.272  0.410  0.422  0.462

Standardized Within-Group Residuals:
        Min          Q1         Med          Q3         Max 
-3.00459176 -0.63717024 -0.06076653  0.47263708  3.55150303 

Number of Observations: 251
Number of Groups: 12 

           R2m       R2c
       0.2123685 0.4788652


###Model II: Model with stand parameters and climate  ###
###Slightly lower AIC compared to model I###
###None of the stand parameters turn out as significant predictor###

Linear mixed-effects model fit by maximum likelihood
 Data: ICP 
       AIC      BIC    logLik
  1527.165 1572.996 -750.5826

Random effects:
 Formula: ~1 | Site_Sp
        (Intercept) Residual
StdDev:    2.035874 7.227987

Correlation Structure: AR(1)
 Formula: ~1 | Site_Sp 
 Parameter estimate(s):
      Phi 
0.7613842 

Fixed effects: iWUE ~ PFT + Age + SoilType + pca_s1 + pca_a1 + pca_a3 
                    Value Std.Error  DF   t-value p-value
(Intercept)      86.85412 12.669466 237  6.855389  0.0000
PFTConifer       12.52487  8.263937   4  1.515606  0.2042
Age              -0.07713  0.148826   4 -0.518274  0.6316
SoilTypeCambisol  0.59838  7.282581   4  0.082166  0.9385
SoilTypeleptosol -7.65834  6.916494   4 -1.107258  0.3303
SoilTypepodzol   -8.85429  5.471761   4 -1.618179  0.1809
SoilTypevertisol  0.17572  5.247793   4  0.033485  0.9749
pca_s1           -2.52740  0.689427   4 -3.665952  0.0215
pca_a1           -0.64567  0.125612 237 -5.140178  0.0000
pca_a3           -0.39759  0.193512 237 -2.054605  0.0410
 Correlation: 
                 (Intr) PFTCnf Age    SlTypC SlTypl SlTypp SlTypv pca_s1 pca_a1
PFTConifer       -0.803                                                        
Age              -0.932  0.666                                                 
SoilTypeCambisol -0.641  0.601  0.428                                          
SoilTypeleptosol -0.612  0.624  0.438  0.527                                   
SoilTypepodzol    0.070 -0.508 -0.132  0.092 -0.113                            
SoilTypevertisol -0.515  0.588  0.225  0.648  0.602  0.052                     
pca_s1            0.050  0.044  0.066 -0.287  0.148 -0.477 -0.156              
pca_a1           -0.006  0.004  0.005  0.003  0.002  0.003  0.005  0.003       
pca_a3            0.001  0.007  0.010 -0.022 -0.011 -0.021 -0.026 -0.004  0.063

Standardized Within-Group Residuals:
       Min         Q1        Med         Q3        Max 
-2.8163705 -0.6981651 -0.1638104  0.5389805  3.8770525 

Number of Observations: 251
Number of Groups: 12 
```

```
##################################################################################
#############################Supplementary text S3################################
##################################################################################
##################################################################################
##################################################################################
###Output from linear mixed  model analyses (LME) for d15N including soil type and age as
fixed factors (see above)####################################################
##################################################################################


##Including only stand parameters in the model##
##They did not result as significant predictors in the model

Linear mixed-effects model fit by maximum likelihood
 Data: ICP
       AIC      BIC    logLik
  535.8694 559.0962 -260.9347

Random effects:
 Formula: ~1 | Site_Sp
        (Intercept)  Residual
StdDev:   0.7575852 0.8222457

Correlation Structure: AR(1)
 Formula: ~1 | Site_Sp
 Parameter estimate(s):
      Phi
0.1391477

Fixed effects: d15N ~ Age + SoilType
                      Value Std.Error  DF    t-value p-value
(Intercept)      -1.9620422 1.3603287 194 -1.4423295  0.1508
Age               0.0035219 0.0201262   6  0.1749904  0.8668
SoilTypepodzol    1.2170226 0.7292056   6  1.6689705  0.1462
SoilTypevertisol -0.0299158 0.7522937   6 -0.0397661  0.9696
 Correlation:
                 (Intr) Age    SlTypp
Age              -0.911
SoilTypepodzol   -0.694  0.413
SoilTypevertisol -0.062 -0.270  0.463

Standardized Within-Group Residuals:
        Min          Q1         Med          Q3         Max
-3.14510897 -0.50460097 -0.04976979  0.56213746  3.51314038

Number of Observations: 204
Number of Groups: 10


           R2m       R2c
    0.2139443 0.5748536


###Adding to the final model presented in the Table 4 soil type and age###
##Soil parameters were not significant predictors in the model

Linear mixed-effects model fit by maximum likelihood
 Data: ICP
       AIC      BIC    logLik
  532.0451 565.2263 -256.0225

Random effects:
 Formula: ~1 | Site_Sp
        (Intercept)  Residual
StdDev:    0.434712 0.8221021

Correlation Structure: AR(1)
 Formula: ~1 | Site_Sp
 Parameter estimate(s):
     Phi
0.138889
Fixed effects: d15N ~ PFT + pca_s1 + sNdep + Age + SoilType
```

```
                      Value Std.Error  DF    t-value p-value
(Intercept)       0.3459480 1.9320438 194  0.1790580  0.8581
PFTConifer       -1.4888226 1.5540633   3 -0.9580192  0.4087
pca_s1            0.3139713 0.2654321   3  1.1828685  0.3221
cNdep             0.0269180 0.1574382   3  0.1709749  0.8751
Age              -0.0136493 0.0179385   3 -0.7608967  0.5021
SoilTypepodzol    0.7145859 0.7686076   3  0.9297148  0.4211
SoilTypevertisol -0.8542589 1.2731924   3 -0.6709582  0.5503
 Correlation:
                 (Intr) PFTCnf pca_s1 sNdep  Age    SlTypp
PFTConifer       -0.895
pca_s1           -0.638  0.774
sNdep            -0.679  0.802  0.956
Age              -0.888  0.667  0.374  0.374
SoilTypepodzol    0.444 -0.723 -0.682 -0.597 -0.317
SoilTypevertisol -0.777  0.873  0.826  0.887  0.428 -0.510

Standardized Within-Group Residuals:
        Min          Q1         Med          Q3         Max
-3.10576182 -0.47165075  0.01063984  0.54893861  3.49073614

Number of Observations: 204
Number of Groups: 10


           R2m      R2c
      0.4460353 0.567083



####################################################################################
############################Supplementary text S4###################################
####################################################################################
####################################################################################
######Running linear mixed  analyses (LME) for iWUE#################################
######with or without Sitka spruce #################################################
####################################################################################
########Running LME model for iWUE with Sitka spruce stands included################
####################################################################################
##All independent variables were centered prior to analysis#########################
##Fixed factors are grouping into conifers and deciduous species (plant functional##
##type, PFT), site (PCA_s) and time (PCA_a) climate variables from PCA analysis,#####
##changes in atmospheric CO2 (ca), spatial (sSdep, sNdep) and annual (aSdep, aNdep)##
##changes in sulphur and nitrogen deposition.#######################################
####################################################################################

## (I) - LME including all the parameters - note that in this case two sites (Covert Wood
and Shobdon were excluded as there are no annual data for Ndep and Sdep, thus the number
of sites included in the analyses is 10 ###


Linear mixed-effects model fit by maximum likelihood
 Data: ICP
       AIC      BIC    logLik
  831.1601 872.3431 -401.5801

Random effects:
 Formula: ~1 | Site_Sp
        (Intercept) Residual
StdDev:    3.624952 4.780661

Correlation Structure: AR(1)
 Formula: ~1 | Site_Sp
 Parameter estimate(s):
      Phi
0.5434811
Fixed effects: iWUE ~ PFT + pca_s1 + pca_a1 + pca_a3 + pca_a2 + sNdep + sSdep +      ca +
aSdep + aNdep

               Value Std.Error  DF  t-value p-value
(Intercept) 80.94442  2.328230 124 34.76651  0.0000
PFTConifer   9.81424  3.209906   5  3.05748  0.0282
```

```
pca_s1      -4.87977  1.123633   5 -4.34285  0.0074
pca_a1      -0.67087  0.170074 124 -3.94458  0.0001
pca_a3      -0.46266  0.253914 124 -1.82211  0.0708
pca_a2      -0.10618  0.219463 124 -0.48383  0.6294
sNdep       -0.59900  0.650696   5 -0.92055  0.3995
sSdep       -1.15434  0.450185   5 -2.56415  0.0504
ca           0.03991  0.087205 124  0.45762  0.6480
aSdep       -0.19915  0.193985 124 -1.02664  0.3066
aNdep        0.11062  0.176846 124  0.62551  0.5328

Correlation:
           (Intr) PFTCnf pca_s1 pca_a1 pca_a3 pca_a2 sNdep  sSdep  ca     aSdep
PFTConifer -0.764
pca_s1      0.170 -0.368
pca_a1      0.068 -0.018 -0.016
pca_a3     -0.008 -0.004  0.011  0.156
pca_a2      0.055 -0.010 -0.002  0.153 -0.012
sNdep       0.025 -0.102  0.767 -0.017  0.012  0.001
sSdep       0.003 -0.172  0.045  0.001  0.000 -0.019 -0.365
ca         -0.150 -0.006 -0.003 -0.116  0.199 -0.046 -0.020  0.024
aSdep      -0.022 -0.003 -0.006 -0.016  0.285  0.105 -0.006  0.007  0.328
aNdep      -0.037  0.003 -0.002  0.067 -0.195 -0.071 -0.012  0.022  0.143 -0.334

Standardized Within-Group Residuals:
       Min         Q1        Med         Q3        Max
-4.2741912 -0.6299001 -0.1422264  0.3886061  2.8677755

Number of Observations: 140
Number of Groups: 10
           R2m       R2c
[1,] 0.6600082 0.7841251

##sSdep is the only significant predictor among the atmospheric deposition parameters. We
run next model, where we include it together with sNdep and ca, which are available for
all sites.


## (II) - LME including climate, ca and mean site Sdep and Ndep as they are available for
all sites - ref. Methods for details. Note that in this case all 12 sites were included in
the model###

Linear mixed-effects model fit by maximum likelihood
 Data: ICP
       AIC      BIC    logLik
  1523.796 1562.575 -750.8978

Random effects:
 Formula: ~1 | Site_Sp
        (Intercept) Residual
StdDev:    3.544995 6.846698

Correlation Structure: AR(1)
 Formula: ~1 | Site_Sp
 Parameter estimate(s):
      Phi
0.7341624
Fixed effects: iWUE ~ PFT + pca_s1 + pca_a1 + pca_a3 + sSdep + ca + sNdep
               Value Std.Error  DF  t-value p-value
(Intercept) 79.67410  2.221875 236 35.85896  0.0000
PFTConifer   9.61270  3.384694   7  2.84005  0.0250
pca_s1      -2.50256  0.751573   7 -3.32976  0.0126
pca_a1      -0.63022  0.126239 236 -4.99228  0.0000
pca_a3      -0.34032  0.196765 236 -1.72960  0.0850
sSdep       -0.60869  0.471488   7 -1.29099  0.2377
ca           0.09443  0.056960 236  1.65778  0.0987
sNdep        0.55355  0.480235   7  1.15267  0.2869
 Correlation:
           (Intr) PFTCnf pca_s1 pca_a1 pca_a3 sSdep  ca
PFTConifer -0.761
pca_s1      0.293 -0.385
pca_a1      0.001 -0.001  0.004
pca_a3      0.000  0.018 -0.015  0.078
```

```
sSdep       0.265 -0.346  0.074  0.001 -0.010
 ca         0.015  0.001 -0.002  0.093  0.193 -0.007
sNdep      -0.104  0.136  0.416 -0.001  0.011 -0.551  0.002

Standardized Within-Group Residuals:
       Min         Q1        Med         Q3        Max
-3.4034691 -0.6107779 -0.1014720  0.5139375  4.3446512

Number of Observations: 251
Number of Groups: 12
           R2m       R2c
[1,] 0.4438774 0.5614461
```

(III) - LME including only significant predictors from model II (note that we included also ca, even though the significance of the t-test was marginal (p<0.1)

```
Linear mixed-effects model fit by maximum likelihood
 Data: ICP
       AIC      BIC    logLik
  1521.644 1553.373 -751.8221

Random effects:
 Formula: ~1 | Site_Sp
        (Intercept) Residual
StdDev:    4.019864 6.914687

Correlation Structure: AR(1)
 Formula: ~1 | Site_Sp
 Parameter estimate(s):
      Phi
0.7400037
Fixed effects: iWUE ~ PFT + pca_s1 + pca_a1 + pca_a3 + ca
               Value Std.Error  DF  t-value p-value
(Intercept) 80.37826  2.303004 236 34.90149  0.0000
PFTConifer   8.22713  3.409152   9  2.41325  0.0390
pca_s1      -2.64961  0.674678   9 -3.92723  0.0035
pca_a1      -0.62891  0.125442 236 -5.01359  0.0000
pca_a3      -0.34253  0.195508 236 -1.75201  0.0811
 ca          0.09427  0.057403 236  1.64228  0.1019
 Correlation:
           (Intr) PFTCnf pca_s1 pca_a1 pca_a3
PFTConifer -0.739
pca_s1      0.306 -0.414
pca_a1      0.001  0.000  0.005
pca_a3      0.002  0.015 -0.020  0.078
 ca         0.017 -0.002  0.000  0.093  0.190

Standardized Within-Group Residuals:
        Min          Q1         Med          Q3         Max
-3.14784262 -0.62576137 -0.08920985  0.53041055  4.13068263

Number of Observations: 251
Number of Groups: 12
           R2m       R2c
[1,] 0.4043793 0.5548325
```

(IV) - LME including only significant predictors from model III. Note that AIC does not significantly change between the two model and from a graphical check (qqplot and residual vs. fitted) we identified the model V as final model and report results in Table 3

```
Linear mixed-effects model fit by maximum likelihood
 Data: ICP
       AIC      BIC   logLik
  1522.332 1550.536 -753.166

Random effects:
 Formula: ~1 | Site_Sp
        (Intercept) Residual
```

```
StdDev:    3.817686 7.155685

Correlation Structure: AR(1)
 Formula: ~1 | Site_Sp
 Parameter estimate(s):
      Phi
0.7560515
Fixed effects: iWUE ~ PFT + pca_s1 + pca_a1 + pca_a3
               Value Std.Error  DF  t-value p-value
(Intercept) 80.32170  2.297150 237 34.96581  0.0000
PFTConifer   8.26064  3.401363   9  2.42863  0.0381
pca_s1      -2.65225  0.673097   9 -3.94037  0.0034
pca_a1      -0.64541  0.124576 237 -5.18086  0.0000
pca_a3      -0.39890  0.191755 237 -2.08024  0.0386
 Correlation:
           (Intr) PFTCnf pca_s1 pca_a1
PFTConifer -0.739
pca_s1      0.306 -0.415
pca_a1     -0.001  0.000  0.005
pca_a3      0.000  0.016 -0.020  0.063

Standardized Within-Group Residuals:
        Min          Q1         Med          Q3         Max
-2.83630783 -0.63549362 -0.07008074  0.53404497  3.70035282

Number of Observations: 251
Number of Groups: 12
          R2m       R2c
[1,] 0.385148 0.5213824



#####################################################################################
###############LME analyses for iWUE without the two Sitka spruce forest stands#####
#####################################################################################

## (I) - LME including all the parameters - note that in this case other two sites (Covert
wood and Shobdon were excluded as there are no annual data for Ndep and Sdep, thus number
of sites is 8 ###


Linear mixed-effects model fit by maximum likelihood
 Data: ICP1
       AIC      BIC    logLik
  671.6546 707.3387 -322.8273

Random effects:
 Formula: ~1 | Site_Sp
        (Intercept) Residual
StdDev:    1.916131  4.34939

Correlation Structure: AR(1)
 Formula: ~1 | Site_Sp
 Parameter estimate(s):
      Phi
0.4562544
Fixed effects: iWUE ~ PFT + pca_s1 + pca_a1 + pca_a3 + sNdep + sSdep + ca + aSdep + aNdep
               Value Std.Error  DF  t-value p-value
(Intercept) 81.73211 1.4989504 102 54.52623  0.0000
PFTConifer   8.83246 2.0881530   3  4.22979  0.0242
pca_s1      -4.76488 0.7154376   3 -6.66010  0.0069
pca_a1      -0.64135 0.1852927 102 -3.46129  0.0008
pca_a3      -0.50598 0.2833109 102 -1.78594  0.0771
sNdep       -0.85590 0.4095735   3 -2.08973  0.1278
sSdep       -1.57566 0.3267597   3 -4.82208  0.0170
ca           0.06280 0.0860291 102  0.73001  0.4671
aSdep       -0.42827 0.2205622 102 -1.94171  0.0549
aNdep        0.08525 0.2083621 102  0.40914  0.6833
 Correlation:
           (Intr) PFTCnf pca_s1 pca_a1 pca_a3 sNdep  sSdep  ca    aSdep
PFTConifer -0.713
pca_s1      0.172 -0.286
```

```
pca_a1      0.093 -0.028 -0.028
pca_a3     -0.022  0.002  0.025  0.036
sNdep      -0.006 -0.082  0.729 -0.035  0.023
sSdep      -0.032  0.004  0.105  0.004  0.001 -0.273
ca         -0.240 -0.014 -0.016 -0.130  0.233 -0.016  0.030
aSdep      -0.074 -0.003 -0.009 -0.120  0.309 -0.001  0.013  0.407
aNdep      -0.045 -0.004 -0.014  0.182 -0.254 -0.023  0.024  0.140 -0.303

Standardized Within-Group Residuals:
        Min          Q1         Med          Q3         Max
-4.66233898 -0.60772496 -0.02984588  0.53163164  2.55540289

Number of Observations: 115
Number of Groups: 8
           R2m       R2c
[1,] 0.7624201 0.8010361
```

(II) - LME including only significant predictors from model I - note that in this case two sites (Covert wood and Shobdon were excluded as there are no annual data for Ndep and Sdep###

```
Linear mixed-effects model fit by maximum likelihood
 Data: ICP1
       AIC     BIC    logLik
  669.7837 697.233 -324.8918

Random effects:
 Formula: ~1 | Site_Sp
        (Intercept) Residual
StdDev:    2.848908 4.401308

Correlation Structure: AR(1)
 Formula: ~1 | Site_Sp
 Parameter estimate(s):
      Phi
0.4740037
Fixed effects: iWUE ~ PFT + pca_s1 + pca_a1 + pca_a3 + sSdep + aSdep
               Value Std.Error  DF  t-value p-value
(Intercept) 81.94220 1.8561987 104 44.14517  0.0000
PFTConifer   8.51365 2.6606580   4  3.19983  0.0329
pca_s1      -3.67297 0.6278045   4 -5.85049  0.0043
pca_a1      -0.65739 0.1769312 104 -3.71548  0.0003
pca_a3      -0.52836 0.2586283 104 -2.04293  0.0436
sSdep       -1.77234 0.4016351   4 -4.41280  0.0116
aSdep       -0.46875 0.1828481 104 -2.56359  0.0118
 Correlation:
           (Intr) PFTCnf pca_s1 pca_a1 pca_a3 sSdep
PFTConifer -0.741
pca_s1      0.267 -0.335
pca_a1      0.054 -0.026 -0.005
pca_a3      0.026  0.005  0.014  0.141
sSdep      -0.029 -0.022  0.463 -0.004  0.005
aSdep       0.020  0.002 -0.007  0.011  0.150  0.007

Standardized Within-Group Residuals:
        Min          Q1         Med          Q3         Max
-4.80905073 -0.55718676 -0.08022529  0.49331243  2.72094981

Number of Observations: 115
Number of Groups: 8
           R2m       R2c
[1,] 0.7115607 0.7967277
```

##We report this model to table 3 as it is the final model where we consider all the atmospheric deposition parameters but a subset of sites (8)

(III) - LME including climate, ca and mean site Sdep and Ndep - ref. Methods for details. Note that in this case all sites were included in the model###

```
Linear mixed-effects model fit by maximum likelihood
 Data: ICP1
       AIC      BIC    logLik
  1250.443 1287.261 -614.2214

Random effects:
 Formula: ~1 | Site_Sp
        (Intercept) Residual
StdDev:    4.240935 4.950081

Correlation Structure: AR(1)
 Formula: ~1 | Site_Sp
 Parameter estimate(s):
      Phi
0.5022159
Fixed effects: iWUE ~ PFT + pca_s1 + pca_a1 + pca_a3 + sSdep + ca + sNdep
               Value Std.Error  DF  t-value p-value
(Intercept) 79.60692  2.118270 197 37.58110  0.0000
PFTConifer   8.90344  3.302905   5  2.69564  0.0430
pca_s1      -2.24491  0.735729   5 -3.05127  0.0284
pca_a1      -0.64768  0.140796 197 -4.60014  0.0000
pca_a3      -0.29698  0.216652 197 -1.37077  0.1720
sSdep       -1.00102  0.496226   5 -2.01726  0.0997
 ca          0.18511  0.039994 197  4.62836  0.0000
sNdep        0.50966  0.465300   5  1.09534  0.3233



Correlation:
           (Intr) PFTCnf pca_s1 pca_a1 pca_a3 cSdep  cca
PFTConifer -0.691
pca_s1      0.316 -0.333
pca_a1      0.003 -0.004  0.004
pca_a3     -0.006  0.018 -0.013  0.025
cSdep       0.270 -0.187  0.059  0.002 -0.007
cca         0.003  0.005 -0.004  0.087  0.312 -0.002
cNdep      -0.137  0.088  0.326 -0.003  0.010 -0.512  0.003

Standardized Within-Group Residuals:
        Min          Q1         Med          Q3         Max
-4.64679468 -0.48380988  0.03740803  0.49086925  2.92905619

Number of Observations: 210
Number of Groups: 10
           R2m       R2c
[1,] 0.5195157 0.7229048


###(IV)- Model with only significant predictors from model III

Fixed effects: iWUE ~ PFT + pca_s1 + pca_a1 + pca_a3 + cSdep + cca
               Value Std.Error  DF  t-value p-value
(Intercept) 79.92494  2.219816 197 36.00520  0.0000
PFTConifer   8.58614  3.480678   6  2.46680  0.0487
pca_s1      -2.50803  0.735687   6 -3.40910  0.0143
pca_a1      -0.64704  0.140430 197 -4.60758  0.0000
pca_a3      -0.29899  0.216085 197 -1.38365  0.1680
cSdep       -0.72263  0.450912   6 -1.60260  0.1601
cca          0.18504  0.039917 197  4.63549  0.0000
 Correlation:
           (Intr) PFTCnf pca_s1 pca_a1 pca_a3 cSdep
PFTConifer -0.688
pca_s1      0.385 -0.385
pca_a1      0.002 -0.003  0.005
pca_a3     -0.004  0.016 -0.016  0.025
cSdep       0.235 -0.166  0.279  0.001 -0.002
cca         0.003  0.005 -0.004  0.087  0.312 -0.001

Standardized Within-Group Residuals:
        Min          Q1         Med          Q3         Max
```

```
-4.54984028 -0.49875782  0.04443618  0.45688679  2.99527689

Number of Observations: 210
Number of Groups: 10
           R2m       R2c
[1,] 0.4896893 0.7228353

##(V)- Only significant predictors from model IV


Linear mixed-effects model fit by maximum likelihood
 Data: ICP1
       AIC      BIC    logLik
  1249.935 1276.712 -616.9676

Random effects:
 Formula: ~1 | Site_Sp
        (Intercept) Residual
StdDev:    5.209536 4.953168

Correlation Structure: AR(1)
 Formula: ~1 | Site_Sp
 Parameter estimate(s):
      Phi
0.4964552
Fixed effects: iWUE ~ PFT + pca_s1 + pca_a1 + cca
               Value Std.Error  DF  t-value p-value
(Intercept) 80.74806  2.420876 198 33.35489  0.0000
PFTConifer   7.73597  3.850666   7  2.00900  0.0845
pca_s1      -2.19373  0.792610   7 -2.76772  0.0278
pca_a1      -0.64295  0.140658 198 -4.57097  0.0000
cca          0.20184  0.037579 198  5.37106  0.0000
 Correlation:
           (Intr) PFTCnf pca_s1 pca_a1
PFTConifer -0.677
pca_s1      0.342 -0.357
pca_a1      0.002 -0.003  0.005
cca         0.004  0.000  0.000  0.083

Standardized Within-Group Residuals:
        Min          Q1         Med          Q3         Max
-4.63255108 -0.48178546  0.05014027  0.45878382  2.95503274

Number of Observations: 210
Number of Groups: 10
           R2m       R2c
[1,] 0.4162629 0.7228476

##THis is the final model shown in Table 3##

####################################################################################
############################Supplementary text S5###################################
####################################################################################
####################################################################################
######Running linear mixed  model analyses (LME) for D18O###########################
######with or without Sitka spruce as shown for iWUE in Table 3#####################
####################################################################################
########Running LME model for D18O with all sites                  #################
####################################################################################
##All independent variables were centered prior to analysis#########################
##Fixed factors are: grouping into conifers and deciduous species (plant functional##
##type, PFT), site (PCA_s) and time (PCA_a) climate variables from PCA analysis,#####
##changes in atmospheric CO2 (ca), spatial (sSdep, sNdep) and annual (aSdep, aNdep)##
##changes in sulphur and nitrogen deposition.#######################################
####################################################################################

## (I) - LME including all the parameters - note that in this case other two sites (Covert
Wood and Shobdon were excluded as there are no annual data for Ndep and Sdep###


Linear mixed-effects model fit by maximum likelihood
 Data: ICP
```

```
       AIC      BIC    logLik
  132.5909 173.7739 -52.29547

Random effects:
 Formula: ~1 | Site_Sp
        (Intercept)  Residual
StdDev:  0.06920099 0.3499774

Correlation Structure: AR(1)
 Formula: ~1 | Site_Sp
 Parameter estimate(s):
      Phi
0.1325319
Fixed effects: (D18O_MB) ~ pca_s1 + pca_a1 + pca_a2 + pca_a3 + ca + PFT + sNdep +
sSdep + aNdep + aSdep
               Value  Std.Error  DF  t-value p-value
(Intercept) 33.09225 0.07350397 124 450.2103  0.0000
pca_s1      -0.28595 0.03328881   5  -8.5901  0.0004
pca_a1      -0.05307 0.01607738 124  -3.3006  0.0013
pca_a2       0.12540 0.02186068 124   5.7364  0.0000
pca_a3       0.04853 0.02496528 124   1.9438  0.0542
ca          -0.00331 0.00584726 124  -0.5668  0.5719
PFTConifer  -0.03618 0.09531956   5  -0.3796  0.7199
sNdep       -0.03650 0.01930717   5  -1.8906  0.1173
sSdep       -0.09380 0.01338924   5  -7.0056  0.0009
aNdep       -0.00399 0.01537827 124  -0.2597  0.7956
aSdep       -0.03362 0.01754163 124  -1.9166  0.0576
 Correlation:
           (Intr) pca_s1 pca_a1 pca_a2 pca_a3 ca    PFTCnf sNdep  sSdep  aNdep
pca_s1      0.140
pca_a1      0.173 -0.048
pca_a2      0.128 -0.008  0.138
pca_a3     -0.068  0.021  0.041  0.011
ca         -0.319  0.010 -0.230  0.053  0.347
PFTConifer -0.728 -0.348 -0.059 -0.032 -0.006 -0.005
sNdep       0.008  0.765 -0.056  0.009  0.043 -0.011 -0.089
sSdep      -0.012  0.044 -0.021 -0.079 -0.016  0.030 -0.165 -0.363
aNdep      -0.068 -0.008  0.082 -0.108 -0.133  0.154 -0.004 -0.025  0.027
aSdep      -0.142  0.004 -0.171  0.174  0.178  0.514 -0.003  0.000  0.004 -0.289

Standardized Within-Group Residuals:
        Min          Q1         Med          Q3         Max
-2.45875536 -0.75755997 -0.09984885  0.72881630  2.26196537

Number of Observations: 140
Number of Groups: 10


           R2m      R2c
[1,] 0.2549247 0.483594



###(II)- Removing n.s. predictors###

Linear mixed-effects model fit by maximum likelihood
 Data: ICP
       AIC      BIC   logLik
  130.2516 162.6097 -54.1258

Random effects:
 Formula: ~1 | Site_Sp
        (Intercept)  Residual
StdDev:   0.1068585 0.3494237

Correlation Structure: AR(1)
 Formula: ~1 | Site_Sp
 Parameter estimate(s):
      Phi
0.1177972
Fixed effects: (D18O_MB) ~ pca_s1 + pca_a1 + pca_a2 + pca_a3 + sSdep + PFT +      aSdep
               Value  Std.Error  DF  t-value p-value
```

```
(Intercept) 33.07803 0.08104600 126 408.1390  0.0000
pca_s1      -0.23807 0.02507908   6  -9.4926  0.0001
pca_a1      -0.05647 0.01537207 126  -3.6735  0.0004
pca_a2       0.12568 0.02150866 126   5.8434  0.0000
pca_a3       0.05466 0.02275689 126   2.4021  0.0178
sSdep       -0.10268 0.01459597   6  -7.0345  0.0004
PFTConifer  -0.05259 0.11117426   6  -0.4731  0.6529
aSdep       -0.02927 0.01335548 126  -2.1916  0.0302
 Correlation: 
           (Intr) pca_s1 pca_a1 pca_a2 pca_a3 sSdep  PFTCnf
pca_s1      0.234                                          
pca_a1      0.097 -0.002                                   
pca_a2      0.130 -0.020  0.173                            
pca_a3      0.038 -0.026  0.163 -0.032                     
sSdep       0.004  0.539 -0.035 -0.070 -0.007              
PFTConifer -0.773 -0.443 -0.058 -0.027 -0.001 -0.218       
aSdep        0.016 -0.002 -0.014  0.135 -0.110 -0.006 -0.002

Standardized Within-Group Residuals:
       Min         Q1        Med         Q3        Max 
-2.6528844 -0.6794309 -0.0438899  0.7180382  2.3333909 

Number of Observations: 140
Number of Groups: 10 


           R2m       R2c
[1,] 0.7234077 0.7470628



###This is the final model including all parameters (but not all sites) included in Table 
4



## (III) - LME including climate, ca and mean site Sdep and Ndep - ref. Methods for 
details. Note that in this case all 12 sites were included in the model###

Linear mixed-effects model fit by maximum likelihood
 Data: ICP 
       AIC      BIC    logLik
  301.5035 343.8089 -138.7517

Random effects:
 Formula: ~1 | Site_Sp
        (Intercept)  Residual
StdDev:   0.3308475 0.4101382

Correlation Structure: AR(1)
 Formula: ~1 | Site_Sp 
 Parameter estimate(s):
      Phi 
0.2424023 
Fixed effects: (D18O_MB) ~ pca_s1 + pca_a1 + pca_a2 + pca_a3 + cSdep + cNdep +      cca + 
PFT 
               Value  Std.Error  DF   t-value p-value
(Intercept) 32.85310 0.15855381 235 207.20472  0.0000
pca_s1      -0.08325 0.05363317   7  -1.55223  0.1645
pca_a1      -0.05072 0.01302027 235  -3.89528  0.0001
pca_a2       0.10799 0.01613795 235   6.69153  0.0000
pca_a3       0.06401 0.02086089 235   3.06848  0.0024
cSdep       -0.06635 0.03361350   7  -1.97404  0.0890
cNdep        0.06487 0.03427151   7   1.89283  0.1003
cca          0.00300 0.00267994 235   1.11988  0.2639
PFTConifer   0.00125 0.24149181   7   0.00520  0.9960
 Correlation: 
           (Intr) pca_s1 pca_a1 pca_a2 pca_a3 sSdep  sNdep  ca
pca_s1      0.293                                             
pca_a1      0.004  0.003                                      
pca_a2     -0.007 -0.003  0.006                               
pca_a3     -0.012 -0.015  0.045  0.052                        
```

```
sSdep       0.265  0.074  0.004 -0.003 -0.011
sNdep      -0.104  0.416 -0.004 -0.001  0.013 -0.551
 ca        -0.005 -0.006  0.063  0.087  0.463 -0.008  0.006
PFTConifer -0.761 -0.385 -0.006  0.002  0.019 -0.348  0.136  0.007

Standardized Within-Group Residuals:
        Min          Q1         Med          Q3         Max
-3.41833526 -0.61578859 -0.03462599  0.65145417  2.43992310

Number of Observations: 251
Number of Groups: 12

           R2m       R2c
[1,] 0.3950397 0.6335177




Linear mixed-effects model fit by maximum likelihood
 Data: ICP
       AIC      BIC    logLik
  301.5035 343.8089 -138.7517

Random effects:
 Formula: ~1 | Site_Sp
        (Intercept)  Residual
StdDev:   0.3308475 0.4101382

Correlation Structure: AR(1)
 Formula: ~1 | Site_Sp
 Parameter estimate(s):
      Phi
0.2424023
Fixed effects: (D18O_MB) ~ pca_s1 + pca_a1 + pca_a2 + pca_a3 + cSdep + cNdep +      cca +
PFT
               Value  Std.Error  DF   t-value p-value
(Intercept) 32.85310 0.15855381 235 207.20472  0.0000
pca_s1      -0.08325 0.05363317   7  -1.55223  0.1645
pca_a1      -0.05072 0.01302027 235  -3.89528  0.0001
pca_a2       0.10799 0.01613795 235   6.69153  0.0000
pca_a3       0.06401 0.02086089 235   3.06848  0.0024
cSdep       -0.06635 0.03361350   7  -1.97404  0.0890
cNdep        0.06487 0.03427151   7   1.89283  0.1003
cca          0.00300 0.00267994 235   1.11988  0.2639
PFTConifer   0.00125 0.24149181   7   0.00520  0.9960
 Correlation:
           (Intr) pca_s1 pca_a1 pca_a2 pca_a3 cSdep  cNdep  cca
pca_s1      0.293
pca_a1      0.004  0.003
pca_a2     -0.007 -0.003  0.006
pca_a3     -0.012 -0.015  0.045  0.052
cSdep       0.265  0.074  0.004 -0.003 -0.011
cNdep      -0.104  0.416 -0.004 -0.001  0.013 -0.551
cca        -0.005 -0.006  0.063  0.087  0.463 -0.008  0.006
PFTConifer -0.761 -0.385 -0.006  0.002  0.019 -0.348  0.136  0.007

Standardized Within-Group Residuals:
        Min          Q1         Med          Q3         Max
-3.41833526 -0.61578859 -0.03462599  0.65145417  2.43992310

Number of Observations: 251
Number of Groups: 12
           R2m       R2c
[1,] 0.3950397 0.6335177


##(IV)- Significant predictors from III###

Linear mixed-effects model fit by maximum likelihood
 Data: ICP
       AIC      BIC    logLik
  302.0017 337.2562 -141.0008
```

```
Random effects:
 Formula: ~1 | Site_Sp
        (Intercept) Residual
StdDev:   0.3834513 0.412155

Correlation Structure: AR(1)
 Formula: ~1 | Site_Sp
 Parameter estimate(s):
      Phi
0.2497009
Fixed effects: (D18O_MB) ~ pca_s1 + pca_a1 + pca_a2 + pca_a3 + sSdep + PFT
               Value  Std.Error  DF   t-value p-value
(Intercept) 32.88463 0.17974915 236 182.94735  0.0000
pca_s1      -0.12495 0.05559784   8  -2.24731  0.0548
pca_a1      -0.05136 0.01295672 236  -3.96400  0.0001
pca_a2       0.10629 0.01605815 236   6.61908  0.0000
pca_a3       0.05282 0.01846147 236   2.86114  0.0046
sSdep       -0.03112 0.03196711   8  -0.97348  0.3588
PFTConifer  -0.06228 0.27268060   8  -0.22839  0.8251
 Correlation:
           (Intr) pca_s1 pca_a1 pca_a2 pca_a3 cSdep
pca_s1      0.372
pca_a1      0.004  0.005
pca_a2     -0.006 -0.002  0.001
pca_a3     -0.009 -0.017  0.018  0.014
cSdep       0.250  0.399  0.002 -0.003 -0.002
PFTConifer -0.758 -0.490 -0.005  0.001  0.014 -0.330

Standardized Within-Group Residuals:
        Min          Q1         Med          Q3         Max
-3.55425663 -0.61987511 -0.04165077  0.61410939  2.41069344

Number of Observations: 251
Number of Groups: 12

          R2m       R2c
[1,] 0.3157569 0.6332246

###Model shown in Table 4####


#####################################################################################
################Running LME model for D18Ow without Sitka spruce stands#############
#####################################################################################


## (I) - LME including all the parameters - note that in this case other two sites (Covert
Wood and Shobdon were excluded as there are no annual data for Ndep and Sdep###

Linear mixed-effects model fit by maximum likelihood
 Data: ICP1
       AIC      BIC    logLik
  119.6407 158.0697 -45.82034

Random effects:
 Formula: ~1 | Site_Sp
         (Intercept) Residual
StdDev: 1.908737e-05 0.362326

Correlation Structure: AR(1)
 Formula: ~1 | Site_Sp
 Parameter estimate(s):
      Phi
0.1063304
Fixed effects: (D18O_MB) ~ pca_s1 + pca_a1 + pca_a2 + pca_a3 + ca + PFT + sNdep +
sSdep + aNdep + aSdep
               Value  Std.Error  DF  t-value p-value
(Intercept) 33.10670 0.06661689 101 496.9715  0.0000
pca_s1      -0.27045 0.02886329   3  -9.3699  0.0026
pca_a1      -0.05935 0.01876551 101  -3.1629  0.0021
pca_a2       0.12741 0.02458774 101   5.1818  0.0000
```

```
pca_a3       0.04584 0.02909066 101   1.5758  0.1182
 ca         -0.00304 0.00669826 101  -0.4540  0.6508
PFTConifer   0.00620 0.08459136   3   0.0733  0.9462
sNdep       -0.04017 0.01656908   3  -2.4243  0.0938
sSdep       -0.08605 0.01328243   3  -6.4788  0.0075
aNdep       -0.00225 0.01963080 101  -0.1144  0.9092
aSdep       -0.02693 0.02167651 101  -1.2422  0.2170
 Correlation:
           (Intr) pca_s1 pca_a1 pca_a2 pca_a3 cca    PFTCnf sNdep  sSdep  aNdep
pca_s1      0.136
pca_a1      0.212 -0.061
pca_a2      0.134 -0.008  0.132
pca_a3     -0.082  0.046 -0.046  0.056
cca        -0.414 -0.006 -0.225  0.111  0.346
PFTConifer -0.660 -0.265 -0.075 -0.036  0.011 -0.013
sNdep      -0.026  0.733 -0.088  0.014  0.065  0.008 -0.064
sSdep      -0.048  0.105 -0.024 -0.080 -0.011  0.033  0.013 -0.268
aNdep      -0.078 -0.028  0.182 -0.103 -0.193  0.141 -0.019 -0.042  0.025
aSdep      -0.205  0.001 -0.218  0.210  0.216  0.551 -0.003  0.018  0.005 -0.291

Standardized Within-Group Residuals:
        Min          Q1         Med          Q3         Max
-2.66802776 -0.73130841 -0.04943957  0.72786789  2.11766886

Number of Observations: 115
Number of Groups: 8
           R2m       R2c
[1,] 0.7002795 0.7002795



#Because among the atmospheric deposition parameters sSdep and sNdep (p<0.1) are
significant predictors in the model, we can now run the model with Cover Wood and Shodden
(but without the two Sitka spruce stands) and including those two parameters and ca again.


## (III) - LME including climate, ca and mean site Sdep and Ndep - ref. Methods for
details. Note that in this case all sites were included in the model###

Linear mixed-effects model fit by maximum likelihood
 Data: ICP1
       AIC      BIC    logLik
  277.7234 314.5416 -127.8617

Random effects:
 Formula: ~1 | Site_Sp
        (Intercept)  Residual
StdDev:   0.3438366 0.4374529

Correlation Structure: AR(1)
 Formula: ~1 | Site_Sp
 Parameter estimate(s):
      Phi
0.2662285
Fixed effects: (D18O_MB) ~ pca_s1 + pca_a1 + pca_a2 + PFT + sSdep + ca + sNdep
               Value  Std.Error  DF   t-value p-value
(Intercept) 32.88029 0.16805620 197 195.65057  0.0000
pca_s1      -0.07574 0.05836531   5  -1.29765  0.2510
pca_a1      -0.05378 0.01496489 197  -3.59373  0.0004
pca_a2       0.10026 0.01839205 197   5.45143  0.0000
PFTConifer   0.06400 0.26200107   5   0.24427  0.8167
sSdep       -0.04711 0.03936761   5  -1.19668  0.2851
 ca         -0.00136 0.00281787 197  -0.48199  0.6303
sNdep        0.05771 0.03691334   5   1.56329  0.1787
 Correlation:
           (Intr) pca_s1 pca_a1 pca_a2 PFTCnf cSdep  cca
pca_s1      0.316
pca_a1      0.006  0.005
pca_a2     -0.007 -0.003  0.017
PFTConifer -0.691 -0.333 -0.007  0.001
cSdep       0.270  0.059  0.004 -0.001 -0.187
cca         0.001  0.000  0.081  0.091 -0.001  0.000
```

```
cNdep      -0.137  0.327 -0.005 -0.001  0.088 -0.512 -0.001

Standardized Within-Group Residuals:
        Min          Q1         Med          Q3         Max 
-3.47849323 -0.61362152 -0.06562452  0.60421418  2.44706786 

Number of Observations: 210
Number of Groups: 10 
           R2m       R2c
[1,] 0.2954597 0.5645048



###(IV)- Final model: including only significant predictors (but keeping PFT)###

Linear mixed-effects model fit by maximum likelihood
 Data: ICP1 
       AIC      BIC    logLik
  274.8804 298.3101 -130.4402

Random effects:
 Formula: ~1 | Site_Sp
        (Intercept) Residual
StdDev:   0.4504732 0.437895

Correlation Structure: AR(1)
 Formula: ~1 | Site_Sp 
 Parameter estimate(s):
      Phi 
0.2674578 
Fixed effects: (D18O_MB) ~ pca_a1 + pca_a2 + PFT 
               Value  Std.Error  DF   t-value p-value
(Intercept) 33.03718 0.19254361 198 171.58285  0.0000
pca_a1      -0.05284 0.01477602 198  -3.57609  0.0004
pca_a2       0.10092 0.01814914 198   5.56064  0.0000
PFTConifer  -0.16256 0.30443495   8  -0.53399  0.6079
 Correlation: 
           (Intr) pca_a1 pca_a2
pca_a1      0.002              
pca_a2     -0.005  0.010       
PFTConifer -0.632 -0.004  0.000

Standardized Within-Group Residuals:
        Min          Q1         Med          Q3         Max 
-3.38748909 -0.63405022 -0.06133657  0.60555201  2.51874803 

Number of Observations: 210
Number of Groups: 10 


           R2m       R2c
[1,] 0.1049902 0.5651648

##Results shown in Table 4


#####################################################################################
#############################Supplementary text S6###################################
#####################################################################################
#####################################################################################
######Running linear mixed model analyses (LME) for d15Nw #######################
######with or without Sitka spruce as shown for iWUE in Table 3#####################
#####################################################################################
########Running LME model for d15Nw  with all sites##############
#####################################################################################
##All independent variables were centered prior to analysis########################
##Fixed factors are: grouping into conifers and deciduous species (plant functional##
##type, PFT), site (PCA_s) and time (PCA_a) climate variables from PCA analysis,#####
##changes in atmospheric CO2 (ca), spatial (sSdep, sNdep) and annual (aSdep, aNdep)##
##changes in sulphur and nitrogen deposition.######################################
#####################################################################################
```

```
##Model I:Including Ndep and ca

Linear mixed-effects model fit by maximum likelihood
 Data: ICP
       AIC      BIC    logLik
  365.1545 397.1125 -171.5772

Random effects:
 Formula: ~1 | Site_Sp
        (Intercept)  Residual
StdDev:   0.5010163 0.8069796

Correlation Structure: AR(1)
 Formula: ~1 | Site_Sp
 Parameter estimate(s):
       Phi
0.01489395
Fixed effects: d15N ~ PFT + pca_s1 + pca_a1 + pca_a3 + sNdep + ca + aNdep

                 Value Std.Error  DF   t-value p-value
(Intercept) -1.1676464 0.2976503 121 -3.922880  0.0001
PFTConifer  -0.0080672 0.4065566   6 -0.019843  0.9848
pca_s1       0.5219081 0.1450911   6  3.597107  0.0114
pca_a1       0.0275127 0.0364118 121  0.755598  0.4514
pca_a3      -0.0341881 0.0592770 121 -0.576751  0.5652
sNdep        0.1402738 0.0778560   6  1.801709  0.1217
ca           0.0025633 0.0111209 121  0.230496  0.8181
aNdep        0.0498688 0.0338781 121  1.472008  0.1436

Correlation:
           (Intr) PFTCnf pca_s1 pca_a1 pca_a3 sNdep  ca
PFTConifer -0.775
pca_s1      0.173 -0.370
pca_a1      0.069 -0.035 -0.033
pca_a3     -0.027 -0.007  0.013  0.034
sNdep       0.029 -0.184  0.842 -0.040  0.024
ca         -0.142 -0.011  0.021 -0.179  0.359  0.008
aNdep      -0.054  0.003 -0.003  0.021 -0.054 -0.011  0.370

Standardized Within-Group Residuals:
        Min          Q1         Med          Q3         Max
-3.46885140 -0.47156397  0.03307331  0.52236707  3.35851437

Number of Observations: 135
Number of Groups: 10

           R2m       R2c
      0.4269688 0.5863962


##Model II - Including only Ndep and climate##

Linear mixed-effects model fit by maximum likelihood
 Data: ICP
       AIC      BIC    logLik
  363.2108 392.2636 -171.6054

Random effects:
 Formula: ~1 | Site_Sp
        (Intercept)  Residual
StdDev:   0.4997899 0.8072875

Correlation Structure: AR(1)
 Formula: ~1 | Site_Sp
 Parameter estimate(s):
       Phi
0.01486507
Fixed effects: d15Nw ~ PFT + pca_s1 + pca_a1 + pca_a3 + sNdep + aNdep
                 Value Std.Error  DF   t-value p-value
(Intercept) -1.1579609 0.2929146 122 -3.953238  0.0001
PFTConifer  -0.0070109 0.4041325   6 -0.017348  0.9867
```

```
pca_s1       0.5212056 0.1442051   6  3.614336  0.0112
pca_a1       0.0290085 0.0356956 122  0.812664  0.4180
pca_a3      -0.0390784 0.0551386 122 -0.708730  0.4798
sNdep        0.1401286 0.0773933   6  1.810603  0.1202
aNdep        0.0469797 0.0313607 122  1.498047  0.1367

Correlation:
           (Intr) PFTCnf pca_s1 pca_a1 pca_a3 sNdep
PFTConifer -0.784
pca_s1      0.177 -0.369
pca_a1      0.045 -0.038 -0.029
pca_a3      0.026 -0.004  0.005  0.107
sNdep       0.030 -0.184  0.842 -0.040  0.022
aNdep      -0.002  0.008 -0.011  0.096 -0.215 -0.015

Standardized Within-Group Residuals:
        Min          Q1         Med          Q3         Max
-3.46237417 -0.46684484  0.02857387  0.52224830  3.36094193

Number of Observations: 135
Number of Groups: 10



###Model II: Including only significant predictor from model II and including Ndep level
as fixed factor. Ndep_lev = low and high Ndep based on the critical loads for the UK (see
Methods for more details)###

Linear mixed-effects model fit by maximum likelihood
 Data: ICP
       AIC      BIC    logLik
  528.4263 551.6531 -257.2131


Random effects:
 Formula: ~1 | Site_Sp
        (Intercept)  Residual
StdDev:   0.5006242 0.8222125

Correlation Structure: AR(1)
 Formula: ~1 | Site_Sp
 Parameter estimate(s):
     Phi
0.139253
Fixed effects: d15Nw ~ PFT + pca_s1 + Ndep_lev
                 Value Std.Error  DF   t-value p-value
(Intercept) -0.7811910 0.4455136 194 -1.753462  0.0811
PFTConifer  -0.1538184 0.4238945   6 -0.362870  0.7291
pca_s1       0.4169451 0.1110545   6  3.754419  0.0095
Ndep_levlow -0.7356892 0.5097808   6 -1.443148  0.1991

Correlation:
            (Intr) PFTCnf pca_s1
PFTConifer  -0.776
pca_s1       0.688 -0.553
Ndep_levlow -0.769  0.406 -0.734

Standardized Within-Group Residuals:
         Min           Q1          Med           Q3          Max
-3.023122900 -0.498834107 -0.004658464  0.553191587  3.488135933

Number of Observations: 204
Number of Groups: 10



##Model III: Including only Years x Ndep level as fixed factor##

Linear mixed-effects model fit by maximum likelihood
 Data: ICP
       AIC      BIC    logLik
  530.6807 553.9075 -258.3404
```

```
Random effects:
 Formula: ~1 | Site_Sp
        (Intercept)  Residual
StdDev:     0.90135 0.7981379

Correlation Structure: AR(1)
 Formula: ~1 | Site_Sp
 Parameter estimate(s):
       Phi
0.08978218
Fixed effects: d15Nw ~ Years * Ndep_lev
                        Value      Std.Error  DF   t-value p-value
(Intercept)            -1.4984354 0.4163013 192 -3.599401  0.0004
Years                   0.0108641 0.0108498 192  1.001324  0.3179
Ndep_levlow             0.6800536 0.5887499   8  1.155081  0.2814
Years:Ndep_levlow       0.0188167 0.0152062 192  1.237441  0.2174
 Correlation:
                   (Intr) Years Ndp_lv
Years              -0.006
Ndep_levlow        -0.707  0.004
Years:Ndep_levlow   0.004 -0.714 -0.001

Standardized Within-Group Residuals:
       Min         Q1        Med         Q3        Max
-3.3920483 -0.4883518 -0.0185723  0.4928521  3.5496385

Number of Observations: 204
Number of Groups: 10



##Model IV - climate and Ndep## Results are reported in the Table 4

Linear mixed-effects model fit by maximum likelihood
 Data: ICP
       AIC      BIC    logLik
  527.1349 550.3617 -256.5674

Random effects:
 Formula: ~1 | Site_Sp
        (Intercept)  Residual
StdDev:    0.464069 0.8221704

Correlation Structure: AR(1)
 Formula: ~1 | Site_Sp
 Parameter estimate(s):
      Phi
0.1391561
Fixed effects: d15N ~ PFT + pca_s1 + sNdep

                 Value Std.Error  DF   t-value p-value
(Intercept) -1.2564004 0.2674005 194 -4.698572  0.0000
PFTConifer  -0.0401130 0.3700437   6 -0.108401  0.9172
pca_s1       0.5140102 0.1319351   6  3.895933  0.0080
sNdep        0.1367236 0.0708935   6  1.928576  0.1020
 Correlation:
           (Intr) PFTCnf pca_s1
PFTConifer -0.785
pca_s1      0.184 -0.376
cNdep       0.037 -0.189  0.844

Standardized Within-Group Residuals:
         Min           Q1          Med           Q3          Max
-3.067836845 -0.506685097 -0.003448838  0.553031791  3.520814256

Number of Observations: 204
Number of Groups: 10

           R2m       R2c
          0.4275086 0.5658329
```

```
#####################################################################################
################Running LME model without Sitka spruce stands########################
#####################################################################################


##Model I: Saturated model with climate and atmospheric factors included as fixed term.##


Linear mixed-effects model fit by maximum likelihood
 Data: ICP1
       AIC     BIC    logLik
  288.3882 315.393 -134.1941

Random effects:
 Formula: ~1 | Site_Sp
        (Intercept)  Residual
StdDev:   0.5139886 0.7676987

Correlation Structure: AR(1)
 Formula: ~1 | Site_Sp
 Parameter estimate(s):
       Phi
0.06829173
Fixed effects: d15Nw ~ PFT + pca_s1 + pca_a1 + pca_a3 + sNdep + aNdep
                 Value Std.Error DF   t-value p-value
(Intercept) -1.2203108 0.3058928 99 -3.989342  0.0001
PFTConifer   0.0036607 0.4419866  4  0.008282  0.9938
pca_s1       0.5002865 0.1515884  4  3.300296  0.0299
pca_a1       0.0037404 0.0378407 99  0.098846  0.9215
pca_a3      -0.0184461 0.0578613 99 -0.318800  0.7506
sNdep        0.1641761 0.0833430  4  1.969885  0.1202
aNdep       -0.0032547 0.0373517 99 -0.087136  0.9307
 Correlation:
           (Intr) PFTCnf pca_s1 pca_a1 pca_a3 cNdep
PFTConifer -0.736
pca_s1      0.193 -0.297
pca_a1      0.044 -0.038 -0.033
pca_a3      0.029  0.008  0.020  0.023
sNdep      -0.008 -0.093  0.789 -0.042  0.024
aNdep       0.000  0.008 -0.017  0.180 -0.242 -0.019

Standardized Within-Group Residuals:
        Min          Q1         Med          Q3         Max
-3.55104018 -0.46649322  0.07335732  0.58231563  2.24564413

Number of Observations: 110
Number of Groups: 8


      R2m       R2c
   0.3832268 0.5741267


##Model II: Including only significant predictor from model I and sNdep#
Linear mixed-effects model fit by maximum likelihood
 Data: ICP1
       AIC      BIC    logLik
  399.4583 421.1146 -192.7292

Random effects:
 Formula: ~1 | Site_Sp
        (Intercept)  Residual
StdDev:   0.4886214 0.7556959

Correlation Structure: AR(1)
 Formula: ~1 | Site_Sp
 Parameter estimate(s):
      Phi
0.1191216
Fixed effects: d15N ~ PFT + pca_s1 + sNdep
                 Value Std.Error  DF   t-value p-value
(Intercept) -1.3099814 0.2798656 155 -4.680751  0.0000
```

```
PFTConifer  -0.0482108 0.4052451   4 -0.118967  0.9110
pca_s1       0.4885342 0.1387827   4  3.520138  0.0244
sNdep        0.1538956 0.0765167   4  2.011269  0.1146
 Correlation: 
           (Intr) PFTCnf pca_s1
PFTConifer -0.737              
pca_s1      0.199 -0.303       
sNdep      -0.003 -0.098  0.789

Standardized Within-Group Residuals:
        Min          Q1         Med          Q3         Max 
-3.32703764 -0.52560566 -0.03014143  0.59893781  2.41937776 

Number of Observations: 163
Number of Groups: 8 


           R2m       R2c
     0.3887468 0.5689547
```

```
##Model III: Including Ndep levels (low and high) in the model and the significant predictor from model I##

Linear mixed-effects model fit by maximum likelihood
 Data: ICP1 
       AIC      BIC    logLik
  401.2295 422.8858 -193.6148

Random effects:
 Formula: ~1 | Site_Sp
        (Intercept)  Residual
StdDev:   0.5532536 0.7557414

Correlation Structure: AR(1)
 Formula: ~1 | Site_Sp 
 Parameter estimate(s):
      Phi 
0.1192319 
Fixed effects: d15N ~ PFT + pca_s1 + Ndep_lev 
                 Value Std.Error  DF    t-value p-value
(Intercept) -0.8184941 0.4890670 155 -1.6735826  0.0962
PFTConifer  -0.1666699 0.4752061   4 -0.3507318  0.7435
pca_s1       0.3812515 0.1286238   4  2.9640818  0.0414
Ndep_levlow -0.7370335 0.5661623   4 -1.3018060  0.2629
 Correlation: 
            (Intr) PFTCnf pca_s1
PFTConifer  -0.695              
pca_s1       0.673 -0.475       
Ndep_levlow -0.769  0.320 -0.674

Standardized Within-Group Residuals:
       Min         Q1        Med         Q3        Max 
-3.2983484 -0.5169073 -0.0246089  0.6050976  2.3439261 

Number of Observations: 163
Number of Groups: 8 

      R2m       R2c
    0.336869 0.5682523
```

```
######Model IV: Including only Years x Ndep level as fixed factor##

Linear mixed-effects model fit by maximum likelihood
 Data: ICP1 
       AIC     BIC    logLik
  402.4857 424.142 -194.2429

Random effects:
 Formula: ~1 | Site_Sp
        (Intercept)  Residual
StdDev:   0.8746482 0.7378314
```

```
Correlation Structure: AR(1)
 Formula: ~1 | Site_Sp
 Parameter estimate(s):
       Phi
0.07757203
Fixed effects: d15N ~ Years * Ndep_lev
                        Value Std.Error  DF   t-value p-value
(Intercept)        -1.5435600 0.4516239 153 -3.417800  0.0008
cYears              0.0190137 0.0108614 153  1.750569  0.0820
Ndep_levlow         0.4017296 0.6387864   6  0.628895  0.5526
cYears:Ndep_levlow -0.0012910 0.0154290 153 -0.083676  0.9334
 Correlation:
                   (Intr) cYears Ndp_lv
cYears             -0.001
Ndep_levlow        -0.707  0.001
Years:Ndep_levlow  0.001 -0.704  0.002

Standardized Within-Group Residuals:
        Min          Q1         Med          Q3         Max
-3.56483329 -0.45974831 -0.03668289  0.58640721  2.51359441

Number of Observations: 163
Number of Groups: 8

           R2m       R2c
         0.04516969 0.6030218


################################End##################################################
```
